# Construction, Deployment, and Usage of the Human Reference Atlas Knowledge Graph for Linked Open Data

**DOI:** 10.1101/2024.12.22.630006

**Published:** 2024-12-23

**Authors:** Andreas Bueckle, Bruce W. Herr, Josef Hardi, Ellen M. Quardokus, Mark A. Musen, Katy Börner

**Affiliations:** 1 Department of Intelligent Systems Engineering, Luddy School of Informatics, Computing, and Engineering, Indiana University, Bloomington, IN, USA; 2 Stanford Center for Biomedical Informatics Research, Stanford University, Stanford, CA, USA

## Abstract

The Human Reference Atlas (HRA) for the healthy, adult body is developed by a team of international, interdisciplinary experts across 20+ consortia. It provides standard terminologies and data structures for describing specimens, biological structures, and spatial positions of experimental datasets and ontology-linked reference anatomical structures (AS), cell types (CT), and biomarkers (B). We introduce the HRA Knowledge Graph (KG) as central data resource for HRA v2.2, supporting cross-scale, biological queries to Resource Description Framework graphs using SPARQL. In December 2024, the HRA KG covered 71 organs with 5,800 AS, 2,268 CTs, 2,531 Bs; it had 10,064,033 nodes, 171,250,177 edges, and a size of 125.84 GB. The HRA KG comprises 13 types of Digital Objects (DOs) using the Common Coordinate Framework Ontology to standardize core concepts and relationships across DOs. We (1) provide data and code for HRA KG construction; (2) detail HRA KG deployment by Linked Open Data principles; and (3) illustrate HRA KG usage via application programming interfaces, user interfaces, and data products. A companion website is at https://cns-iu.github.io/hra-kg-supporting-information.

## Introduction

The multimodal, three-dimensional (3D) Human Reference Atlas (HRA)^1^ aims to map the healthy, adult human body across scales—from the whole body to the single cell and biomarker levels. Data from different sources (organs, technologies, and labs), many built with standard operating procedures (SOPs, https://humanatlas.io/standard-operating-procedures), need to be integrated. The HRA Knowledge Graph (KG) defines and provides the core data structures that are used to store, link, and query HRA data.

KGs are widely used to interlink data about relevant entities within a specific domain or task. The Google Knowledge Graph (https://blog.google/products/search/introducing-knowledge-graph-things-not) supports Google Search with its billions of searches processed daily. Major online shopping retailers such as Amazon^2^ use knowledge graphs to organize products, searches, and media items. KGs across domains are structured using vocabularies, e.g., Friend of a Friend (FOAF, http://xmlns.com/foaf/spec), Simple Knowledge Organization System (SKOS, https://www.w3.org/2004/02/skos), and Music Ontology (http://musicontology.com). Plus, there exist collaborative efforts for publishing structured data on the web. For example, https://schema.org promotes the structured representation for data on the web and is used in applications from Google, Microsoft, and Pinterest to create data-driven web experiences. An overview of other commonly used vocabularies is available on https://www.w3.org/wiki/TaskForces/CommunityProjects/LinkingOpenData/CommonVocabularies.

In the biomedical domain, ontologies are widely used to structure data, which is of high relevance to HRA KG construction. For example, the National Center for Biomedical Ontology (NCBO) BioPortal provides easy access to 1,168 ontologies and the EMBL-EBI Ontology Lookup Service (OLS) supports 267 ontologies. The Uber-anatomy ontology (Uberon, https://www.ebi.ac.uk/ols4/ontologies/uberon)^3^ is a cross-species ontology representing body parts, organs, and tissues, primarily focused in vertebrates. The Cell Ontology (CL, https://obofoundry.org/ontology/cl.html)^4^ is also a cross-species ontology, but it focuses on classifying and describing cells. These two ontologies are linked data, so we can determine assertions such as kidney nerve cell (CL:1000606) has location (RO:0002100) kidney (UBERON:0002113) from the knowledge represented in the ontologies.

Ontologies are an indispensable part of generating, using, and maintaining KGs as they enable unifying nomenclature across assay types, organs, donors, teams, and consortia. A recent publication by He et al.^5^, featuring the HRA, shows how ontologies can be used to model, integrate, and reason over previously siloed clinical, pathological, and molecular kidney data for precision medicine. It highlights the development of the precision medicine metadata ontology (PMMO) to integrate dozens of variables between the Kidney Precision Medicine Project (KPMP, https://www.kpmp.org)^6,7^ and Chan Zuckerberg Initiative (CZI) CELLxGENE (CxG) data (https://cellxgene.cziscience.com). It then shows specific use cases in detecting healthy vs. acute kidney infection (AKI)/chronic kidney disease (CKD) disease states in cells supported by PMMO, Kidney Tissue Atlas Ontology (KTAO), and the HRA’s CCF Ontology, described in a related publication^8^.

Biomedical KGs use and interlink multiple ontologies to store and query data. For example, the Unified Medical Language System (UMLS)^9^ “metathesaurus” contains approximately 3.4 million biomedical concepts, updated every 6 months in May and November and is derived from other biomedical terminologies and ontologies. The Petagraph KG^10^ uses the UMLS metathesaurus to integrate biomolecular datasets and connects them to approximately 200 cross-referenced ontologies to support exploration of gene variant epistasis as well as biological assertions with reduced dimensionality, and to link relevant features to chromosome position and chromosomal neighborhoods. The Human BioMolecular Atlas Program (HuBMAP, https://hubmapconsortium.org)^11,12^ Unified Biomedical Knowledge Graph^1^ (UBKG) connects HuBMAP experimental data to ontologies. The Scalable Precision Medicine Open Knowledge Engine (SPOKE,https://spoke.ucsf.edu)^13,14^ processes 41 databases (53 million edges) and 11 ontologies to create an integrated graph with user access via a Representational State Transfer (REST) application programming interface (API). Petagraph, HuBMAP, and SPOKE use the Neo4J graph platform (https://neo4j.com). Efforts like BioCypher (https://biocypher.org)^15^ enable the rapid construction and maintenance of KGs at lower cost. This also addresses the lack of reusability and integrability, where KGs are built manually for a specific task, and, as a result, do not adhere to Findable, Accessible, Interoperable and Reusable (FAIR)^16^ principles. KGs can be used to extract knowledge across constantly evolving ontologies and data in various states of accessibility (private and public).

In this paper, we present the HRA KG v2.2, which uses 10 ontologies to interlink 34 anatomical structures (AS), cell types (CT), plus biomarkers (B) tables (see Box 1), 71 3D Reference Objects for organs,, 22 Functional Tissue Units (FTUs)^17^, 11,698 single-cell (sc) datasets, and other HRA Digital Objects (DOs, see Results > HRA Digital Objects), together with the HRA API that supports efficient queries using REST, JavaScript, TypeScript, Angular 17+, or Python 3.6+ code plus several interactive user interfaces (UIs).

Specifically, we present open code and infrastructure to construct the HRA KG out of disparate data across tabular/non-tabular and nested/flat HRA DOs while ensuring processed data conforms to 5-star Linked Open Data (LOD)^18^ principles. The HRA KG data can be accessed via content negotiation from https://lod.humanatlas.io and dynamically queried via its SPARQL (see Box 1) endpoint at https://lod.humanatlas.io/sparql.

### Related Work

The goal of the HRA KG is to make the increasing amount of expertise and biomedical knowledge inside HRA DOs (see Box 1 and Results > HRA Digital Objects) programmatically accessible. Similarly, with the growing diversity, volume, and complexity of biomedical data (including scientific literature) in recent years, KGs have been used extensively to extract, represent, and make programmatically available knowledge otherwise obscured in scientific papers. Recently, SemOpenAlex^30^ provided more than 26 billion RDF triples describing scholarly knowledge. The SemOpenAlex Explorer (https://semopenalex.org) enables non-programmer access to the SemOpenAlex KG. The Leibniz Information Centre for Science and Technology University Library in Hannover (Germany) publishes the Open Research Knowledge Graph^31^ (ORKG, https://orkg.org) alongside an AI tool called ORKG Ask (https://ask.orkg.org). The tool allows researchers without programming expertise to browse data-driven comparisons and thematic paper collections. KGs have also been used to facilitate mining biological entities out of 29,000,000 PubMed abstracts^32^. The creators performed author name disambiguating, added funding data via the NIH ExPORTER (https://reporter.nih.gov/exporter), and collected educational and professional backgrounds of author via Open Researcher and Contributor ID (ORCID, https://orcid.org) and MapAffil^33^. Various biological areas have been captured in KGs: RNA-KG^34^ federates entities related to ribonucleic acid (RNA) from 60 public resources with the goal of aiding RNA therapy development. The authors preprocessed each disparate data source, built a metagraph using ontology terms for biomolecular entities and their interactions, then made the graph queryable with SPARQL endpoint and allowed the user to download data products in different formats. The open-source RTX-KG2^35^ allows building a KG across 70 data sources, incl. UMLS, the Semantic MEDLINE (https://pubmed.ncbi.nlm.nih.gov) Database (SemMedDB)^36^, ChEMBL^37^, Reactome^38,39^, DrugBank^40^, and the Small Molecule Pathway Database (SMPDB). It includes provenance and scientific references where applicable. The aforementioned UBKG (https://ubkg.docs.xconsortia.org) connects related concepts from biomedical ontologies and vocabularies. It combines the concept of assertions, i.e., the chaining of two entities and a relationship into a subject-predicate-object relationship, with the UMLS to build connections between sets of assertions. Petagraph^10^ (https://github.com/TaylorResearchLab/Petagraph) is built using UBKG to integrate over 20 large multi-omics human and mouse genomic datasets. It leverages 180+ ontologies, and annotation resources that support a diversity of genomics data types, like STRING (https://string-db.org), Reactome (https://reactome.org)^38^, and GENCODE (https://www.gencodegenes.org). The goal of Petagraph is to harmonize omics data for rapid feature selection and hypothesis generation. It contains 32 million nodes and 118 million relationships. Like UBKG, Petagraph uses Neo4j and does not allow queries via SPARQL. For custom queries, the maintainers need to add an endpoint. Note also that a license is needed to use UMLS (https://www.nlm.nih.gov/databases/umls.html).

An essential part of building the HRA KG (and others) is to link entities in it to ontologies (see Results > HRA KG Construction, Deployment, and Usage) to make the data in it FAIR^16^ and enable connections to other units of biomedical knowledge. In a study^41^ of the the reuse of study metadata in ClinicalTrials.gov, values in 302,091 trial records were tested for their adherence to expected data types and use terms from biomedical ontologies. Among other issues, the authors found that ontologies are not the only vocabulary used for search-related fields, e.g., condition and intervention. Further, almost 50% of the conditions were not annotated with Medical Subject Headings (MeSH, https://www.ncbi.nlm.nih.gov/mesh) terms. Ontologies can be used to provide controlled, unified vocabulary across entities in a KG. This facilitates the aggregation of research resources, such as results from clinical trials from disparate sources, terms for human anatomy, and cell type typologies. The Center for Expanded Data Annotation and Retrieval (CEDAR)^42,43^ and its Workbench aims to unify how metadata for biomedical datasets are created, annotated, and shared to facilitate data sharing and acceleration of discovery. An important element increasingly used to support standardization efforts are Common Data Elements (CDEs), which define specific questions and their permissible answers. Although CDEs offer a solid conceptual basis for interoperability, there is no broadly accepted format for their serialization or exchange.

The application, extension, and validation of ontologies is a separate research area. To make annotation with ontology-aligned terms easier for researchers and developers, validators are used for building graphical representations of ontology views and user-provided hierarchies, e.g., for the HRA and the Human Developmental Cell Atlas (https://www.humancellatlas.org/dca)^44^. The Stimulating Peripheral Activity to Relieve Conditions (SPARC, https://sparc.science) effort constructs its own vocabulary from ontologies in relevant domains, e.g., anatomy and physiology^45^. This SPARC Vocabulary is applied in segmentation and annotation tools so that ontological terms can be applied to research data. To address the issue that these annotations happen in experimental data, where not all terms and relationships may be represented in community ontologies, researchers can expand SPARC Vocabulary by managing terms via InterLex (https://scicrunch.org/resolver/RRID:SCR_016178), a lexicon for biomedical terms used by the National Institute of Diabetes and Digestive and Kidney Diseases (NIDDK) Information Network (https://dknet.org), among others. Relatedly, the SPARC Connectivity Knowledgebase of the Autonomic Nervous System (SCKAN)^46^ provides SPARC data on the autonomic nervous system (ANS) and provides origins, terminations, and routing of ANS projections for improved neuromodulation devices and bioelectronic medicine for nervous system diseases. In this case, SCKAN presents distilled connectivity knowledge by experts, published literature, textbooks, and SPARC scientific data. SCKAN is used to automatically generate anatomical and functional connectivity maps on the SPARC portal (https://sparc.science/apps/maps?type=ac). Like SCKAN^46^, the Data Distillery Knowledge Graph (DDKG, https://dd-kg-ui.cfde.cloud/), on the other hand, focuses on extracting essential knowledge out of disparate data sources in the Common Fund Data Ecosystem (CFDE, https://info.cfde.cloud/). It aims to facilitate better integration and reuse of CF data to accelerate discoveries in biomedical research by extending Petagraph’s schema to integrate over 30 large genomics datasets in collaboration with NIH Common Fund efforts, such as HuBMAP, the Cellular Senescence Network (SenNet, https://data.sennetconsortium.org)^47^, Gabriella Miller Kids First Pediatric Research (Kids First, https://kidsfirstdrc.org), 4D Nucleome (https://4dnucleome.org)^48^, the Genotype-Tissue Expression (GTEx, https://gtexportal.org/home)^49^ Project, and the Library of Integrated Network-Based Cellular Signatures (LINCS, https://lincsproject.org/LINCS). Like Petagraph, it is scalable for new genomics data types and cross-dataset analyses and includes development of user-friendly interfaces (API, UI) for secure and efficient querying. It contains 40 million nodes and 300 million relationships. A set of use cases is available at https://dd-kg-ui.cfde.cloud/use_cases.

## Results

### HRA Digital Objects

HRA DOs come in diverse formats, such as ASCT+B tables^22^, 3D Reference Objects, and OMAPs^27^. Each DO has a type, name, and version. For example, *asct*-*b/kidney/v1.5* indicates *asct*-*b* as the type, *kidney* as the name, and *v1.5* as the version. These DOs were provided by SMEs (see Box 1), then reviewed and validated throughout the data acquisition process.

As an example, ASCT+B tables are complex data structures. When constructing a table, SMEs are asked to crosswalk AS, CT, and B terms to ontology terms so they can be mapped to the HRA. ASCT+B tables make it possible for anatomists, surgeons, and other experts to digitize knowledge about the cells and biomarkers in healthy tissue; however, parsing of unprocessed ASCT+B tables is not advisable, as detailed validation and additional enrichment are required before the data can be used in HRA construction, see details in the Result > HRA KG Construction section.

**Figure 1.**
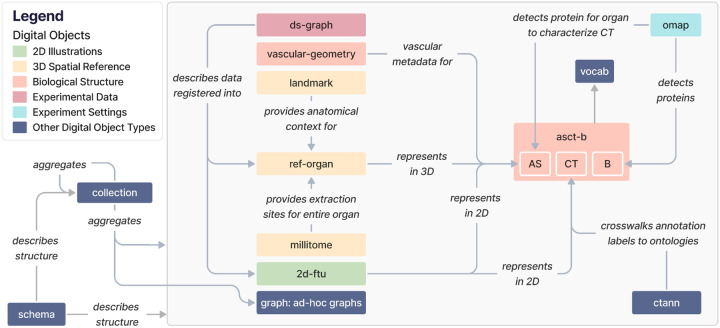
The 13 DO types in the HRA KG and how they relate to each other. Note that we replaced underscores in edge labels with blank spaces for legibility. For class names, we added a blank space between CamelCased class names. Entity-relationship diagrams are provided on the companion website at https://cns-iu.github.io/hra-kg-supporting-information.

A complete list of DO types in HRA v2.2 is provided in Table 1. DOs are available in a variety of formats on the LOD server at https://lod.humanatlas.io (see Methods > HRA KG Construction and Deployment > Data Processing Pipeline). Figure 1 illustrates high-level relationships among the 13 DO types.

The high-level relationships between DO types are as follows:

2D Illustrations (green): *2d*-*ftu* DOs illustrate both AS (because FTUs are AS) and the CT in them, based on experimental data. *2d*-*ftu* DOs can be downloaded in their processed form or as scalable vector graphics (SVG), Portable Network Graphics (PNG), or Adobe Illustrator (AI) files.

3D Spatial Reference (yellow): *ref*-*organ* DOs get anatomical context from *landmark* DOs, and *millitome* DOs provide extraction sites for an entire organ that a *ref*-*organ* DO represents. *ref*-*organ* and *landmark* DOs can be downloaded as GLB files, the binary format of glTF (Graphics Library Transmission Format or GL Transmission Format, see https://www.khronos.org/gltf). *millitome* DOs can be downloaded in the JavaScript Object Notation for Linked Data format (JSON-LD, https://json-ld.org).

Biological Structure (warm pink): The *asct*-*b* DO type plays a central role for multiple other DO types, see Box 1 and Table 1. *vascular*-*geometry* DOs provide vascular metadata for *asct*-*b* DOs. Both can be downloaded in their raw distributions as comma-separated values (CSV) files.

Experimental Data (muted pink): *ds*-*graph* DOs describe experimental datasets mapped into a *ref*-*organ*. They can be downloaded as JSON-LD files.

Experiment Settings (bright blue): *omap* DOs enable detection of proteins and cell types and are thus connected to AS and Bs in *asct*-*b* DOs. *omap* DOs can be downloaded as CSV or Microsoft Excel files (XLSX).

Other DO types (dark blue):

CTann crosswalks (*ctann*) map manual and machine learning annotations for cell types from different cell type annotation tools and workflows to the ASCT+B tables. *ctann* and *omap* DOs allow us to map experimental datasets, represented as *ds*-*graphs*, into the HRA. *ctann* DOs can be downloaded as CSV files. *vocab* DOs are referenced by *asct*-*b*, *omap*, *ctann*, and *ref*-*organ* DOs to annotate AS, CT, and Bs with ontology terms and can be downloaded as Web Ontology Language files (OWL, https://www.w3.org/OWL). *graph* DOs are ad-hoc graphs that can reference any other DO type as needed, depending on their function and scope, and can thus have any download format available for the referenced DOs. All current *graph* DOs in the HRA KG are listed in Supplemental Table 1. *collection* DOs aggregate multiple DOs and can be downloaded as YAML files. The *collection* type combines other DOs to allow end users to create customized configurations for their particular needs. Importantly, the HRA itself is a collection DO (https://lod.humanatlas.io/collection/hra/latest/). All current collections in the HRA KG are listed in Supplemental Table 2. Finally, the *schema* DO type describes the structure of all 12 other DO types plus their metadata and can be downloaded in a variety of formats, including YAML, PNG, and SVG.

### HRA KG Construction, Deployment, and Usage

The HRA KG represents major DOs of the HRA v2.2, including 36 ASCT+B tables, 23 OMAPs, 22 2D FTUs, 71 3D Reference Objects (plus two whole body models with all organs for male/female and a crosswalk from 3D AS to ontology terms), see https://apps.humanatlas.io/dashboard/data. In December 2024, the HRA KG had 10,064,033 nodes, 171,250,177 edges, and a size of 125.84 GB. The size of the 71 3D Reference Objects (GLB files, https://lod.humanatlas.io/ref-organ) in HRA v2.2 is 301 MB. In addition, the data covers anatomical landmarks which are used in the HRA Registration User Interface (RUI)^19^ to facilitate tissue block placement in 3D Reference Objects; these are available at https://lod.humanatlas.io/landmark. As of HRA v2.2, there are landmarks for 59 out of 65 3D Reference Objects. Together, they are 261 MBs large.

**Figure 2.**
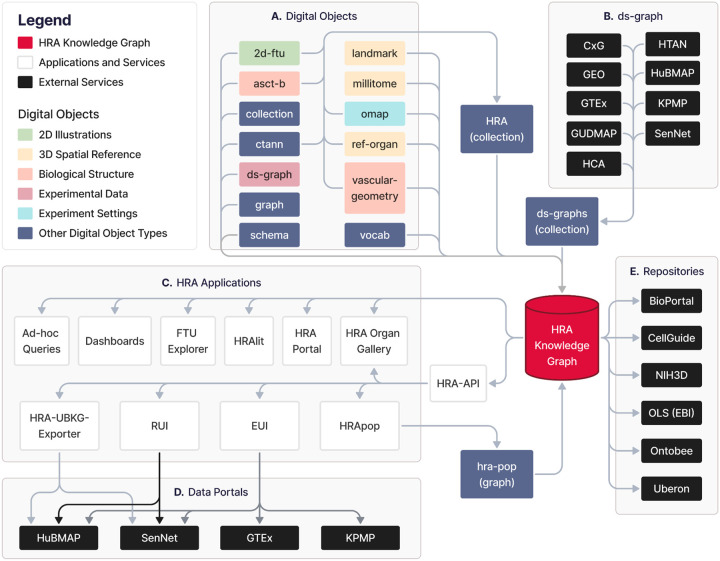
DOs (A), experimental datasets (B), HRA applications (C), data portals (D), and external services (E) form the ecosystem around the HRA KG. Note that the HRA KG is able to serve all existing versions of the HRA and HRA DOs. **(A)** 13 types of HRA DOs fully describe the HRA. They are represented as a HRA KG collection. 703 individual DOs go through a 3-step process of normalization, enrichment, and deployment via the hra-do-processor (https://github.com/hubmapconsortium/hra-do-processor), where they are transformed from raw data in miscellaneous file formats to an RDF graph. Thus, the HRA KG integrates the knowledge from these DOs. Together, the HRA DOs form the HRA collection, which is the graph representation of the HRA. All 13 DO types are described in detail in the HRA DOs section above. The normalization, enrichment, and deployment are described in the HRA Construction subsection and Methods > HRA KG Construction and Deployment > Data Processing Pipeline below. **(B)** Graph representations of external experimental datasets from various sources with mean B expression values and cell population data, resulting in the *ds*-*graph* DO type. Experimental data from various portals is mapped into the HRA via one or a combination of multiple methods, such as 3D tissue registration^19^, CL^4^ aligned Azimuth^23^, CellTypist^24,25^, and popV^26^ annotations, OMAPs^27^ for spatial proteomic data, or via ontology crosswalks to ASCT+B tables. These portals include the CZI CxG portal (https://cellxgene.cziscience.com), the Gene Expression Omnibus (GEO, https://www.ncbi.nlm.nih.gov/geo), the GTEx^49^ Portal (https://gtexportal.org/home), the GenitoUrinary Developmental Molecular Anatomy Project (GUDMAP, https://www.atlas-d2k.org/gudmap)^60^, the Human Cell Atlas (HCA, https://www.humancellatlas.org/data-portal)^61^, the Human Tumor Atlas Network (HTAN, https://humantumoratlas.org/explore)^62^, the HuBMAP^11,12^ Data Portal (https://portal.hubmapconsortium.org), the KPMP^6,7^ Kidney Tissue Atlas (https://atlas.kpmp.org), and the SenNet^47^ Data Portal (https://data.sennetconsortium.org/search). **(C)** HRA applications and services use the HRA KG as their main data backend through the HRA API or via a SPARQL endpoint (https://lod.humanatlas.io/sparql). The HRA Portal (https://humanatlas.io)^1^ and Dashboard (https://apps.humanatlas.io/dashboard) provide usage and data statistics about the HRA by querying the HRA KG. The HRA API provides the HRA UIs with access to Uberon and CL IDs for AS, CT, and B as well as spatial entities for tissue blocks and organs^8^ for the RUI and Exploration User Interface (EUI)^19^. For example, when using the EUI to select AS, CT, and B terms (on the left side of the UI), counts are retrieved from those relationships that are curated from multiple graphs. Since all graphs are in the RDF format, it is feasible to query across multiple graphs seamlessly without modifying the source graphs. HRA Cell Type Populations (HRApop) provide cell types and mean biomarker expressions for experimental datasets mapped to the HRA. The FTU Explorer (https://apps.humanatlas.io/ftu-explorer)^63^ accesses CL IDs for cells and HUGO Gene Nomenclature Committee (HGNC, https://www.genenames.org)^64^ IDs for biomarkers via the HRA KG. Ad-hoc queries to retrieve counts and access DOs from the HRA KG are easily possible via the SPARQL endpoint. HRAlit data^65^ (https://github.com/x-atlas-consortia/hra-lit) connects 136 DOs from HRA v1.4 to 583,117 experts, 7,103,180 publications, 896,680 funded projects, and 1,816 experimental datasets. The HRA Organ Gallery in virtual reality (VR)^66^ utilizes the HRA KG to show predicted cell types in tissue blocks in immersive, 3D space. The primary API at https://apps.humanatlas.io/api has programming language-specific client libraries in JavaScript, TypeScript, Angular 17+, and Python 3.6+. These client libraries are published to common code package managers, including NPM (https://www.npmjs.com) and PyPi (https://pypi.org), and they wrap the API calls into simple function calls to use from code, making HRA data easy to use from software development environments. A full list of client libraries is available at https://humanatlas.io/api. A set of example Python Notebooks are provided at https://github.com/x-atlas-consortia/hra-api/tree/main/notebooks. **(D)** The HRA KG is used in several **data portals**, including some from which *ds*-*graph* DOs are being extracted: HuBMAP, SenNet, GTEx, and KPMP. For example, the HRA-UBKG Exporter (https://github.com/x-atlas-consortia/hra-ubkg-exporter) is used to make HRA data available for HuBMAP Data Portal services (https://portal.hubmapconsortium.org), such as Uberon^3^ aligned organ pages (e.g., https://portal.hubmapconsortium.org/organ/lung), and AS search and filter functionality. Links to publicly accessible instances of UIs using the HRA KG on data portals are provided in Supplemental Table 3. **(E)** Several **external services** serve and/or use the HRA KG data: NCBO Bioportal and Ontobee host the HRA CCF Ontology^8^ (see Box 1) at https://bioportal.bioontology.org/ontologies/CCF and https://ontobee.org/ontology/CCFO. The OLS provides a collection of HRA DOs for validation at https://www.ebi.ac.uk/ols4/ontologies/hra. CellGuide (https://cellxgene.cziscience.com/cellguide) utilizes the ASCT+B tables to identify and present to their users canonical biomarkers for cell types. Finally, the NIH3D platform by the National Institute of Allergy and Infectious Diseases (NIAID) hosts all 71 3D Reference Objects for organs in the HRA v2.2 (https://3d.nih.gov/collections), plus two United files with all organs combined. 22 2D FTU illustrations are at https://bioart.niaid.nih.gov/discover?collection=2.

To make raw as well as processed HRA DOs available in a programmatic manner as RDF graphs, the HRA KG sits at the center of the HRA data ecosystem and serves as the primary database for the HRA, see Figure 2.

### Metagraph

**Figure 3.**
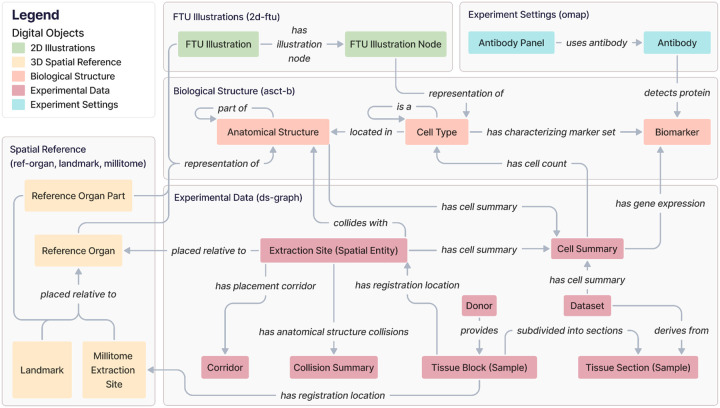
This metagraph illustrates high-level relationships within the HRA KG.

HRA DO types can be aggregated into five thematic subgraphs: Spatial Reference, FTU Illustrations, Experiment Settings, Biological Structure, and Experimental Data. Figure 3 presents the HRA KG metagraph, which depicts the higher-order relationships among these interconnected subgraphs. The Spatial Reference subgraph (yellow) specifically is presented and explored in detail in a prior publication^8^.

The **Biological Structure** subgraph anchors all other components. It contains the AS, CTs, and Bs together with their ontological relationships. AS and CT can have self-loops, where an AS can be part of another AS, creating a partonomy, and a CT can be a subclass of another CT in a typology.

The **Spatial Reference** subgraph represents the 3D CCF used to accurately position (3D) *Reference Organs*, anatomical landmarks, and millitomes (see Table 1) within the human body. This subgraph links to the Biological Structure to provide the 3D anatomical context for the AS.

Similarly, the **FTU Illustrations** subgraph uses the Biological Structure subgraph to enrich its 2D FTU Illustrations and the FTU Illustration Nodes within it with the ontology-aligned naming for FTUs and CT.

The **Experimental Data** subgraph focuses on experimental Datasets generated from assay analyses performed on Donor Tissue Blocks (Samples). These are assigned an Extraction Site with the RUI^19^ based on their anatomical origins to provide a location within the CCF. Since HRA v1.2, extraction sites are *placed relative to* Reference Organs; note that this is also a change in terminology, which we used to call *has_placement*^8^. All possible alternative locations of an extraction site given its intersection(s) with one or multiple 3D AS are captured in a Corridor. Systematic whole-organ registration is available as a Millitome, which defines a set of connected extraction sites *placed relative to* a Reference Organ. This subgraph also accommodates derived data computed from the assay results and extraction sites, such as (a) Cell Summaries, which provide cell type populations and mean gene expression values for specific cell types and their associated datasets and 3D extraction sites, and (b) Collision Summaries, which identify AS that overlap with the registered tissue blocks inside a 3D extraction site.

Finally, the **Experimental Settings** subgraph catalogs Antibody Panels via OMAPs^27^, capturing details of specific Antibodies used to detect particular biomarkers.

### HRA KG Construction

The 13 HRA DO types described above come from SMEs, who contribute their knowledge of anatomy, antibodies, pathology, and experimental procedures via disparate data sources. Manually curated and experimental datasets from diverse sources need to be mapped to the HRA and standard ontologies, normalized to a standard format (e.g., unification of term labels), and enriched (e.g., linked to existing ontologies in support of causal reasoning). We developed a software tool called **hra-do-processor** (https://github.com/hubmapconsortium/hra-do-processor) to normalize and enrich these DOs, then deploy them as RDF graphs. A catalog of these graphs is available on the HRA KG LOD server at https://lod.humanatlas.io. A complete list of code pieces used for HRA KG construction, deployment, and usage is shown in Supplemental Table 4. The HRA KG is constructed twice a year, coinciding with the HRA release cycle^1^ (see release notes at https://humanatlas.io/overview-training-outreach#release-notes).

The data processing pipeline uses a sequence of five steps to convert different raw datasets into the HRA KG: normalization, enrichment, deployment, finalization, and serving. Implementation details for the data processing pipeline are provided in the Methods > HRA KG Construction and Deployment > Data Processing Pipeline section.

#### Normalization:

This initial step ensures that all incoming data is transformed into a consistent format that aligns with the predefined schema. For example, unnormalized, raw ASCT+B tables are available via the ASCT+B Reporter (https://apps.humanatlas.io/asctb-reporter). A normalized example is provided in the form of an ASCT+B record in our format in Supplemental Figure 1.

#### Enrichment:

This step converts the normalized data to RDF graph format and enriches it with related relationships, entities, and metadata from external resources, either from ontologies like Uberon or CL or via APIs like HGNC.

#### Deployment:

Once the data is enriched, it is prepared for use in downstream applications or for access by end users. This stage involves organizing the data into its final distribution formats and setting up the correct file system directory structure.

#### Finalization:

This step involves generating the necessary metadata and landing pages for web publication, e.g., https://lod.humanatlas.io/asct-b/eye/latest leads to the most recently published ASCT+B table for the eye. In addition, this stage includes building the SPARQL database that will be uploaded to the web for users to access at https://lod.humanatlas.io/sparql.

#### Serving:

Data processed in the previous steps, including raw DO data, processed data products, HTML pages, metadata, and the SPARQL database, are made available online at https://lod.humanatlas.io. The data is regularly updated and synchronized, either during scheduled releases or when updates occur, to ensure that the most current version is always available.

### HRA KG Deployment

The HRA KG LOD server at https://lod.humanatlas.io provides metadata for processed HRA DOs as Data Catalog Vocabulary (DCAT) datasets (https://www.w3.org/TR/vocab-dcat-3) in five different graph formats, including JSON-LD. Every DO has a PURL (see Box 1), such as https://purl.humanatlas.io/asct-b/lymph-node/v1.4. Raw data files are hosted on a global content delivery network (CDN), e.g., https://cdn.humanatlas.io/digital-objects/ref-organ/knee-female-right/v1.3/assets/3d-vh-f-knee-r.glb.

The next section details how HRA DOs, e.g., ASCT+B tables, can be accessed and used in the JSON-LD format via Python. We deliver the graph data via Amazon Web Services (AWS, https://aws.amazon.com).

### HRA KG Usage

Users access the HRA KG via UIs, APIs, and data products on https://lod.humanatlas.io to answer biomedical questions. A list of all HRA applications that use the HRA KG is provided in Supplemental Table 3. A list of publications and aliases used throughout HRA applications per HRA DO is provided in Supplemental Table 5.

The HRA KG makes it possible to access harmonized, high-quality reference and experimental data in standard data formats. Three widely used queries are detailed here: (1) retrieve AS-CT-B records from the ASCT+B tables, (2) compute mean biomarker expression values for cell types across datasets in HRApop, and (3) query the HRA KG to achieve two types of predictions: predict cell type populations for extraction sites, and predict 3D registration corridors for a given cell type population, see HRA user stories 1–2 in a related publication^1^.

To simplify HRA KG usage, we use the https://grlc.io service to make a set of canned SPARQL queries available to execute as simple web requests. Internally, the service creates an OpenAPI specification (https://swagger.io/specification) that advertises the available queries. We provide a user-friendly interface to these queries at https://apps.humanatlas.io/api/grlc. This deployment was inspired by the PubMed MeSH SPARQL Explorer at https://id.nlm.nih.gov/mesh/query.

### Get records from ASCT+B tables

The HRA KG API makes it easy to retrieve AS-CT-B records, properties, or counts for one or multiple organs. Exemplary queries and links to resulting data are provided here. Documentation of the OpenAPI specification is at https://apps.humanatlas.io/api/grlc/hra.html. Annotated screenshots of the query interface with instructions on how to run the queries or download resulting data can be found at the companion website at https://cns-iu.github.io/hra-kg-supporting-information/#how-to-run-queries-via-our-openapi-spec.

#### Get ASCT+B counts for all tables:

Retrieve the number of unique AS, CT, and B terms across all ASCT+B tables via this query for the latest HRA release:

- https://apps.humanatlas.io/api/grlc/hra.html#get-/as-ct-b-counts

#### Returns:

A table with the number of unique AS, CT, B in the latest version of the HRA.

Retrieve the number of unique AS, CT, and B terms across all ASCT+B tables for all HRA releases:

- https://apps.humanatlas.io/api/grlc/hra.html#get-/as-ct-b-counts-all-versions

#### Returns:

A table with the number of unique AS, CT, B across all versions of the HRA (not just the latest one).

Note that the only difference between the two queries is the added *FROM HRA:* statement, which limits the SPARQL search pattern of the first query to the latest HRA collection at https://purl.humanatlas.io/collection/hra.

#### Get ASCT+B records for one organ:

Given the PURL of an *asct*-*b* DO, retrieve ASCT-B records from the ASCT+B table:

- https://apps.humanatlas.io/api/grlc/hra.html#get-/asctb-in-table.

#### Returns:

A table with one row per AS-CT-B record in the specified table, in the format: AS, CT, B labels, and the AS, CT, B ontology ID (if crosswalked, otherwise it returns a temporary ID).

Exemplary Python code that uses this endpoint is provided on the companion website at https://cns-iu.github.io/hra-kg-supporting-information#basic-usage. The user can choose from common data formats (CSV or JSON) for the response via the *Accept* header.

#### Get ASCT+B records for all organs:

Retrieve individual records for all ASCT+B tables (rather than just counts or records for one organ):

- https://github.com/x-atlas-consortia/hra-pop/blob/main/queries/hra/asctb-records.rq

#### Returns:

A table with one row per AS-CT-B record for all ASCT+B tables, in the format: AS, CT, B labels, and the AS, CT, B ontology ID (if crosswalked, otherwise it returns a temporary ID). Because the result of this query is large (1,048,576 rows and a total of 325 MB) and takes longer to run, it has not been deployed via https://grlc.io. Rather, the query response has been preprocessed and is available for download as a zipped CSV file on GitHub: https://github.com/x-atlas-consortia/hra-pop/blob/main/output-data/v0.11.1/reports/hra/asctb-records.csv.zip.

An exemplary Jupyter Notebook on how to run a SPARQL query against the HRA KG is at https://cns-iu.github.io/hra-kg-supporting-information/#notebook-to-query-the-hra-knowledge-graph-kg.

### Retrieve Mean Biomarker Expression Values for Cell Type(s)

The hra-pop *graph* (https://lod.humanatlas.io/graph/hra-pop/latest) in the HRA KG contains mean B expression values for CTs inside datasets. To compute mean B expressions, scanpy^67^, numpy^68^, and anndata^69^ are used. Concretely, scanpy’s to *rank_gene_groups()* method assigns mean biomarker expressions. We normalize gene names with a lookup table from Ensembl Release 111^64^ (https://www.ensembl.org/index.html) to HGNC v2023-09-18^70^ (https://www.genenames.org). Both the code to compute mean B expressions and the look-up table from Ensembl to HGNC are linked in Supplemental Table 1 under the entry for the hra-pop *graph*.

#### Retrieve mean biomarker expression values for a given cell type across organs:

Retrieve all experimental datasets from HRApop that contain a canonical CT plus biomarker expression values with this query:

- https://apps.humanatlas.io/api/grlc/hra-pop.html#get-/datasets-with-ct

#### Returns:

A table with atlas datasets that have the given CT. There is one CT-BM expression per row. The response includes the dataset source (which portal it was downloaded from), the dataset ID (must be an Internationalized Resource Identifier [IRI, https://www.w3.org/International/O-URL-and-ident.html]), the organ, the donor sex, the tool that assigned the CT, the CT ontology ID (same for every row as provided by user), a human-readable CT label, the number of cells of the given type in the dataset, a B ontology ID, and finally, the mean expression value for all Bs that characterize this CT computed via CTann tools (see Box 1) in HRApop^1^. If multiple CTann tools were used for the same CT and dataset, multiple rows are provided to show the different mean Bs.

### Predict Cell Type Populations and Locations

Seven user stories^1^ have been identified for the HRA based on interviews with more than 40 atlas architects working on human atlases, see Supplemental Table 6. While all user stories are directly supported by HRA UIs and the HRA API for users with little to no programming experience, the HRA KG can also be queried directly in support of these user stories. Supplemental Table 6 also lists sample queries that help experts retrieve knowledge from DOs, identify processed data of interest, and run analyses to answer biomedical questions. Here, we detail the queries that support the US#1 (Predict cell type populations) and #2 (Predict spatial origin of tissue samples).

#### Predict cell type populations (US#1):

The HRA KG is used access the HRApop graph (https://lod.humanatlas.io/graph/hra-pop/latest) with cell type populations for AS, datasets, and extraction sites to improve the accuracy of annotations for sc-transcriptomics and sc-proteomics datasets. A demonstration application is available at https://apps.humanatlas.io/us1.

The user provides an extraction site using the RUI. The extraction site is then posted to the HRA API (https://apps.humanatlas.io/api/#post-/hra-pop/rui-location-cell-summary), which returns a predicted cell summary given the 3D collisions of the extraction site with AS inside the 3D Reference Object for which the extraction site was defined. Under the hood, the API queries a graph inside the HRA KG (https://cdn.humanatlas.io/digital-objects/graph/hra-pop/v0.11.1/assets/atlas-as-cell-summaries.jsonld) to retrieve a predicted cell summary for the extraction site using the SPARQL query at https://github.com/x-atlas-consortia/hra-api/blob/main/src/library/hra-pop/queries/as-weighted-cell-summaries.rq.

#### Predict spatial origin of tissue samples (US#2):

For the inverse case, the cell type populations from the HRApop graph mentioned above can be used to predict the 3D location of datasets with unknown spatial origin. A demonstration application is available at https://apps.humanatlas.io/us2.

The user provides a cell summary for a given dataset whose origin is uncertain or unknown (beyond basic metadata such as the organ and the tool used to assign different CT). The application features an optional dropdown menu to select the organ and tool. Supported organs and tools for the dropdown menu are available via the HRA API endpoints at https://apps.humanatlas.io/api/hra-pop/supported-organs and https://apps.humanatlas.io/api/#get-/hra-pop/supported-tools, respectively. These endpoints run SPARQL queries under the hood: https://github.com/x-atlas-consortia/hra-api/blob/main/src/library/hra-pop/queries/supported-reference-organs.rq (to get the reference organs supported by HRApop) and https://github.com/x-atlas-consortia/hra-api/blob/main/src/library/hra-pop/queries/supported-tools.rq (to get the tools).

Once the user provides the cell type population of the dataset of unknown spatial origin, the application posts this population to an endpoint in the HRA API (https://apps.humanatlas.io/api/hra-pop/cell-summary-report), which takes a minimum of a CT ID and a column for the percentage of that CT in the dataset.

Under the hood, the HRA API runs the SPARQL query at https://github.com/x-atlas-consortia/hra-api/blob/main/src/library/hra-pop/queries/select-cell-summaries.rq, which returns a listing of the most similar AS, datasets, and extraction sites (by cosine similarity).

## Discussion

This paper detailed the construction, deployment, and usage of the HRA KG to make HRA DOs available as LOD (see Box 1). The HRA provides a CCF (see Box 1) with standard terminologies and data structures for describing specimens, biological structures, and spatial positions of experimental datasets and ontology-linked reference AS, cell types, and biomarkers. It makes it possible to map and integrate anatomical, cellular, and molecular data of the human body in 3D. It also enables researchers and clinicians to consistently annotate, compare, and understand biological structures and analysis results across tissues. The HRA KG offers a structured, interconnected data representation of the HRA, incorporating dozens of highly curated reference datasets and supporting different HRA UIs.

The HRA KG makes it possible to access HRA data efficiently and to ask biological questions via programmatic queries. Researchers can leverage the KG to enrich their assay data for deeper insights, while clinicians can use it to explore biological questions, such as identifying the types and populations of epithelial cells in the human eye. The HRA KG is composed of multiple named graphs, each focusing on a specific part of the atlas, such as biological structures or spatial references. The HRA *collection* is a collection of DOI’d DOs (ASCT+B Tables, 3D Reference Objects, OMAPs, etc.) that make up the core of the HRA at each release. When processed, it compiles to a (large) RDF graph and is hosted by the HRA KG at https://purl.humanatlas.io/collection/hra.

The HRA uses a KG (as opposed to a relational database) to ensure (1) **Flexibility**. The schema of the HRA KG can be extended as needed when new organs or HRA DO data types become available (as opposed to a rigid schema that would need to be chosen for a relational database). (2) **Easy extension**. The HRA KG can be easily extended with DOs for new organs. Existing DOs can easily be updated. In a relational database, one would need a set of new tables. Many HRA DOs, such as 3D Reference Objects, are non-tabular and highly nested, which is highly challenging to model in relational databases. (3) **Support for disparate data**. HRA DOs take on many forms. For example, ASCT+B tables capture AS, CTs in those structures, and the Bs that characterize them; they are linked to OMAP and Antibody Validation Report (AVR)^27^ tables (tabular data), 2D images and 3D models (graphic assets), as well as dataset graphs such as HRAlit^65^ and HRApop (highly-nested). A relational database would make it necessary to choose a schema for each of these DO types. A KG enables integration of many different DO types. (4) **Answering biological questions across HRA DO types**. The KG structure makes it easier to programmatically answer questions across multiple DOs for one entire organ via graph queries (e.g., the ASCT+B table for the kidney and the 3D Reference Object for the female, left kidney). In a relational database, this would necessitate a set of new tables that would need to be carefully created with foreign keys and relationships to support the kind of dynamic graph-based queries readily available in SPARQL. (5) **Deployment as 5-Star LOD**. RDF graphs can be imported into triple stores in their native format and easily be queried together with connected biomedical ontologies (genes, proteins, cells, anatomy), which are also published as RDF. Existing HRA KG queries bridge CL^4^, Provisional Cell Ontology (PCL)^71^, Uberon^3^, HGNC^64^, and HRA nodes, properties, and relationships stored or imported from their respective graphs.

### Limitations & Future Work

The current HRA KG has a number of known limitations that will be addressed in future HRA releases:

#### Automation:

While many parts of the HRA KG construction process are automated, collecting and providing DOs in their original form (CSVs, GLBs, SVG, etc.) is still a manual process involving human labor. In future releases, we aim to employ machine learning algorithms^17,72–80^ to speed up data segmentation and annotation, using human expertise to review (not hand-compile) DOs.

#### Build time:

At present, building the HRA KG from unprocessed DOs using code in https://github.com/hubmapconsortium/hra-kg takes about 13 hours on a local Linux server with 256GB RAM and 20 cores. As new DO types are added and HRApop and HRAlit grow, we will need to optimize the construction process by optimizing KG structures, using parallelization, and optimizing normalization and enrichment code (some libraries are particularly slow for certain DO types). Preliminary results from one experiment showed a nearly one-third reduction in execution time, demonstrating the potential of parallelization in optimizing KG construction.

#### Reduce ASCTB-TEMP terms:

As of HRA v2.2, 221 CTs across 36 ASCT+B tables do not yet exist in CL or PCL; instead they have an ASCTB-TEMP expert provided label. GitHub issues have been submitted for all and the EBI team is adding these terms to existing ontologies. As of December 2024, a total of 141 CT were added to CL^4^, and 461 CT were added to PCL^71^ by ASCT+B table editors. Additionally, 126 AS terms were added to Uberon^3^.

#### Data modeling:

To be most useful to the HRA KG, each new DO type must have a LinkML schema (see Box 1), normalization code, and enrichment code to transform the raw data into useful, queryable information. As new use cases are identified, the HRA structure and canned queries will be revised and expanded. Currently, HRAlit is being served via a relational database. Knowledge modeling is underway to create a HRAlit KG and to properly connect it to the HRA KG, which will allow users to query peer-reviewed literature and funded awards for entities in the HRA KG.

#### Ease of use:

Retrieving data from KGs requires experience writing SPARQL queries, which few clinicians and biomedical researchers possess. The HRA KG comes with canned queries at https://apps.humanatlas.io/api/grlc/ as well as Jupyter Notebooks (see companion website at https://cns-iu.github.io/hra-kg-supporting-information). Going forward, we will create an HRA Developer Portal to help train and provide resources to users learning how to use the HRA KG. Additionally, since KGs offer access to structured data, we are exploring the possibilities of utilizing modern large language models (LLMs) to allow users to ask questions in prose. An LLM, enhanced by retrieval-augmented generation (RAG), could be used to build a chatbot for easy natural language queries that are informed by the knowledge in the HRA KG. With the research space around LLMs changing and the possibilities surrounding them expanding, we are monitoring this space for use cases and opportunities. Further, we will continue to grow the HRA KG by federating datasets, assays, samples, and donors from an ever increasing number of portals and consortia. Finally, we are working on an HRA KG Explorer UI, which will allow users to browse the KG via the web to quickly identify, select, and download HRA DOs of interest in all available graph formats. This will enable easy access to the HRA KG to users without experience writing code, making API requests via https://grlc.io, or running SPARQL queries.

## Methods

All data and code needed to construct, deploy, and use the HRA KG are available at https://github.com/hubmapconsortium/hra-kg. A full list of all data and code is available in Supplemental Table 4.

### HRA KG Construction and Deployment

The hra-do-processor (https://github.com/hubmapconsortium/hra-do-processor) is built around three main components: the schema, the data processing pipeline, and the web infrastructure. The following sections detail each of these components.

### Schema

Well-defined data schemas are crucial for ensuring data consistency, interoperability, and validation in data management and analysis. SOPs that define the schema make it easier for others to contribute to and use the HRA data. LinkML^29^is a flexible and user-friendly schema language designed to create effective data models and validation tools to ensure input data adheres to a defined schema. Entity relationship diagrams of core HRA schemas explain the relationships between different HRA DOs, see examples on the companion website at https://cns-iu.github.io/hra-kg-supporting-information/#mermaid-diagrams.

### Data Processing Pipeline

The HRA KG data processing pipeline has five key steps:

#### Normalization:

The hra-do-processor loads and parses the disparate source data produced by diverse SMEs and transforms it into a standardized linked-data representation. For example, in the case of *asct*-*b* DOs, the source data comes from Google Sheets exported as CSV files; during normalization, the hra-do-processor reads this tabular structure and converts it into the tree structure shown in Supplemental Figure 1. An exemplary YAML file is provided at https://github.com/cns-iu/hra-kg-supporting-information/blob/main/docs/intermediary_format.yaml. We chose YAML as the standard file format for the normalized data due to its simplicity, readability, interoperability with JSON, and high level of support in LinkML. By converting the LinkML schema into a JSON-Schema file, we can easily validate the translated YAML data to be sure it adheres to the defined schema and our automated ingestion code is correctly implemented.

#### Enrichment:

After the source data is translated into YAML and validated, enrichment begins by converting the validated data into OWL-based statements. The LinkML framework offers tools that facilitate the transformation of tree-structured data into OWL constructs, including class and property declarations, as well as class instances (or individuals) (see Supplemental Figure 2). We chose OWL as the data representation for the enrichment step due to its robust capabilities for rich data expression, its ability to embed semantic meaning, and its seamless integration with LinkML. LinkML provides direct support for OWL by allowing schema elements to include the type of OWL constructs, making it easy to map the data into a semantically rich ontology structure.

The enrichment process continues by integrating additional information from reference ontologies such as Uberon, FMA, and CL, as well as authoritative databases like Research Resource Identifier (RRID, https://www.rrids.org) and the Antibody Registry API (https://www.antibodyregistry.org) to retrieve metadata (label, description) about antibodies, for which there is only an RRID in the raw HRA DO for OMAPs. The goal is to enhance the initial data gathered from SMEs with more detailed, authoritative information. In *asct*-*b* DOs, many data points reference Uberon and CL terms. We enrich these terms by retrieving supplementary information from the corresponding ontologies, including class hierarchies, labels, definitions, synonyms, database references, and visual depictions. For example, we identified the standard label for a CL:0002306 as “epithelial cell of proximal tubule,” which is categorized under the broader class “meso-epithelial cell.” These details, which were absent from the original dataset, add valuable context. The end result is a semantically enriched dataset that not only preserves the original data but also extends it with additional context, relationships, and meaning.

#### Deployment:

HRA data is used by many different tools and user communities. HRA UIs like the EUI and RUI^19^ use JSON-LD, which is best when using the data directly and imperatively (i.e., in a programming language using *for* loops), e.g., Python and JavaScript have native support for handling JSON and has semantics built in.

The Blazegraph (https://blazegraph.com) SPARQL server uses Terse RDF Triple Language (Turtle, https://www.w3.org/TR/rdf12-turtle); additionally, the Turtle format helps developers write SPARQL queries to the HRA KG by making its triple structure explicit and showing possible subjects, predicates, and objects. Older semantic web tools use RDF/Extensible Markup Language (XML, https://www.w3.org/TR/rdf-syntax-grammar), N-Triples (https://www.w3.org/TR/n-triples/), and N-Quads (https://www.w3.org/TR/n-quads). Additionally, Robot (http://robot.obolibrary.org/convert.html), Apache Jena(https://jena.apache.org/documentation/io/), and RDF I/O technology (RIOT) use XML for reifying graphs. HRA KG data is preprocessed in those formats for it to be readily usable by others. Publishing all these formats streamlines the content negotiation process later (see Box 1) when different applications access the published HRA KG on the LOD server at https://lod.humanatlas.io, which can then immediately deliver the HRA data in the correct format. The tool also prepares the metadata that accompanies the graph data.

The deployment process includes setting up the file system by creating directories to organize and store output files for deployment. The hra-do-processor then copies files and data assets into their designated folders as a preparation for the next step. Data and metadata for each DO are pre-converted into the formats previously mentioned.

#### Finalization:

In the deployment step, data and metadata for each DO are converted and exported. Finalization derives additional files across all DOs, including metadata catalogs, latest versions of each DO, HTML landing pages to navigate the HRA KG and view DOs, and an indexed and optimized database file for the Blazegraph SPARQL server accessible at https://lod.humanatlas.io/sparql. The database contains the latest version of every DO, every version of the HRA collection, and a metadata catalog that contains metadata for every version of every DO in the HRA KG.

#### Serving:

To make the processed data widely accessible, AWS is used to serve the HRA KG as linked open data, employing three of its core services: S3, ECS and CloudFront for data storage, computation, and content delivery, respectively. Implementation details are provided in the next section.

### Web infrastructure

*Amazon S3 (Simple Storage Service)* is a highly scalable data storage service to store and retrieve data. The HRA KG uses S3 to store the content from the local deployment directories, including the Blazegraph database file. By syncing these local directories with an S3 storage, the data is securely stored and readily available for content delivery.

*Amazon ECS (Elastic Container Service)* is a fully managed container service to run applications in Docker containers for a highly scalable and reliable environment for our computation needs (https://www.docker.com). For HRA, a Blazegraph instance is run within an ECS container. The ECS container periodically checks the S3 storage for an updated Blazegraph database file. When a newly built Blazegraph file is detected, ECS will seamlessly update the Blazegraph server to ensure that the latest data is available for querying.

*Amazon CloudFront* is a global Content Delivery Network (CDN) designed to accelerate the distribution of content by caching copies at multiple serving locations around the world. The HRA KG uses CloudFront to create a URL fabric that caches and serves content from S3 storage to ensure fast and reliable access for users, regardless of their geographical location. The content stored in S3 is made publicly available through URLs like https://cdn.humanatlas.io/digital-objects and https://lod.humanatlas.io. Additionally, CloudFront provides advanced content negotiation features through Amazon CloudFront functions, enabling the dynamic handling of URLs starting with https://purl.humanatlas.io and https://lod.humanatlas.io. Content negotiation allows the web infrastructure to serve data in different formats based on user needs, whether a user requires RDF, XML, JSON, or another format. The PURL returns HRA DO *data* based on the *Accept* header of the request: human users get redirected to the LOD Server, machines to JSON or RDF versions. The LOD Server returns HRA DO *metadata* based on the *Accept* header: Human users get HTML, machines get JSON or RDF. Moreover, CloudFront also acts as an intermediary for the SPARQL endpoint hosted by Blazegraph within ECS by making it accessible at https://lod.humanatlas.io/sparql.

### Updates since the CCF.OWL paper (HRA v1.2 / CCF.OWL v2.0.1)

The specimen, biological structure, and spatial ontologies in support of a HRA (v1.2) using CCF v2.0.1 were introduced in a prior publication^8^. Starting with HRA v2.0, published in December 2023, the CCF Ontology (v3.0) is separated from the HRA collection. CCF v3.0 is a DO of type (*vocab*) and the HRA collection is a DO of type (*collection*). Before then, it was a graph, essentially the HRA *collection* plus the CCF Ontology. Now the HRA collection references the CCF and is compiled from a collection of curated HRA DOs and is hosted by the HRA KG at (https://purl.humanatlas.io/collection/hra/v2.2). This change was necessary to establish a boundary between the framework for creating atlases, the CCF, and a specific atlas, the HRA.

### Other Ontologies

The HRA KG includes other reference ontologies at https://lod.humanatlas.io/vocab (e.g., Uberon and CL) so they can be queried together in an efficient manner. Table 2 lists all ontologies that are included in the HRA KG together with their version numbers.

## Supplementary Material

1

## Figures and Tables

**Table 1.** Different DO types used in the HRA KG, describing their purposes and the data they contain plus SOPs detailing the construction of these DOs.

Digital Object	Description
**Reference data**	
asct-b	Represents an ASCT+B table, see Box 1. Contains detailed knowledge about human body parts in a hierarchical order, explaining the organization of AS, the CT in each AS, and the Bs that distinguish each CT^22^. An SOP is available^50^.
2d-ftu	Provides 2D illustrations of FTU structures in an organ, with image assets and cell annotations that assign proper labels and identifiers based on CL for each image segment. An SOP is available^51^.
ref-organ	Represents a 3D Reference Object, see Box 1. Provides 3D models of human organ structures, complete with accurate size and position data, to support the creation of a comprehensive 3D model of the human body, with each 3D model object carefully annotated with a proper label and an identifier from the Uberon and FMA ontologies. Multiple SOPs are available^52,53^.
landmark	Provides 3D model shapes representing features near organs of interest (e.g., an artery or pelvis bone near a kidney) to help experts accurately orient themselves when registering tissue blocks into a 3D Reference Object.
vascular-geometry	Provides detailed geometry information on the human blood vascular system which captures key attributes of different vessels such as diameter and length, population, sample size, and reference to the source of data. Multiple SOPs are available^54–57^.
millitome	Provides data about cutting tissue samples using a millitome device. An SOP is available^58^.
ctann	Represents a crosswalk, see Box 1. Translates cell type labels or abbreviations from sc/snRNAseq analysis tools, specifically Azimuth^23^, CellTypist^24,25^, and popV^26^ into standardized terms in CL. The translation quality is measured using standard predicates, such as *exact match* and *narrow match* to ensure consistent data harmonization across sc/snRNAseq analyses.
omap	Represents an OMAP, see Box 1. Reduces the costs of conducting cell imaging experiments. OMAPs^27^ contain a panel of antibodies designed to target specific proteins for identifying CTs, AS, cell states, or cell membrane staining within organs, based on actual experimental projects. An SOP is available^59^.
schema	Describes the structure, i.e., the schema, of the normalized form of a single DO type, its metadata, or shared concepts between DOs.
vocab	Contains various reference ontologies and vocabularies that hold standard concepts and relationships used to construct the DOs. These DOs are typically external biomedical ontologies like CL and Uberon and provide a convenient mechanism for querying reference ontologies alongside HRA-curated DOs.
collection	Combines multiple DOs to create a collection of data. The HRA itself is defined in the HRA KG as a curated *collection* of DOs in each release version.
**Experiment data**			
ds-graph	Provides sample registration information submitted by consortium members in HuBMAP or other efforts, including accurate sample sizes and positions. When combined with *ref-organ* data, this information helps create 3D visual tissue sample placements. Additionally, the sample information is linked to datasets from researchers’ assay analyses that offer deeper insights into the tissue samples.
graph	Contains externally created RDF (see Box 1) graph data that are useful for HRA use cases.

**Table 2.** Ontologies used in the HRA KG as of HRA v2.2.

Name	Description	Version Number	URL	Main Website
CCF	Common Coordinate Framework Ontology^8^	3.0	httDs://Durl.humanatlas.io/vocab/ccf	https://humanatlas.io/ccf-ontoloay
CL	Cell Ontology^4^	2024-09-26	https://purl.humanatlas.io/vocab/cl	https://obophenotvpe.aithub.io/cell-ontoloav/
FMA	Foundational Model of Anatomy^20,21^	5.0.0	https://purl.humanatlas.io/vocab/fma	http://si.washinaton.edu/proiects/fma
HGNC	HUGO Gene Nomenclature Committee^64^	2024-03-04	https://purl.humanatlas.io/vocab/hanc	https://www.aenenames.ora/
HRAVS	HuBMAP Research Attributes Value Set	2.5.3	https://purl.humanatlas.io/vocab/hravs	https://bioportal.bioontoloav.ora/ontoloaies/HRAVS
LMHA	Cell Ontology for Human Lung Maturation (LungMAP Human Anatomy)^81^	1.4	https://purl.humanatlas.io/vocab/lmha	https://bioportal.bioontoloav.ora/ontoloaies/LUNGMAP_H_CELL
PCL	Provisional Cell Ontology^71,82^	2024-07-11	https://purl.humanatlas.io/vocab/pcl	https://aithub.com/obophenotvpe/provisional_cell_ontoloav
RO	OBO Relation Ontology (httDs://doi.ora/10.5281/zenodo.32899)	2024-04-24	https://purl.humanatlas.io/vocab/ro	https://aithub.com/oborel/obo-relations
Uberon	Uberon Multi-species Anatomy Ontology^3^	2024-11-25	https://purl.humanatlas.io/vocab/uberon	https://obophenotvpe.aithub.io/uberon/
VCCF	Vasculature Common Coordinate Framework^22,83^	2024-02-23	https://purl.humanatlas.io/vocab/vccf	https://aithub.com/hubmapconsortium/hra-vccf

## Data Availability

The primary server for the HRA KG v2.2 is at https://lod.humanatlas.io. The SPARQL endpoint to query the HRA KG is at https://lod.humanatlas.io/sparql. The HRA API (https://apps.humanatlas.io/api) supports programmatic access to the HRA KG and exemplary queries are available via the companion website at https://cns-iu.github.io/hra-kg-supporting-information/. BioPortal hosts (1) the HRA at https://bioportal.bioontology.org/ontologies/HRA (mirror of https://purl.humanatlas.io/collection/hra/v2.2) and (2) the CCF Ontology at https://bioportal.bioontology.org/ontologies/CCF (mirror of https://lod.humanatlas.io/vocab/ccf). OLS hosts the latest versions of both the HRA and CCF at https://www.ebi.ac.uk/ols4/ontologies/hra and https://www.ebi.ac.uk/ols4/ontologies/ccf, respectively. OLS provides both a web-based GUI for users and programmatic access via the OLS REST API (https://www.ebi.ac.uk/ols4/help), enabling the HRA and CCF to be accessed using the same standard interface as other ontologies. The NIH3D platform by NIAID hosts all 71 3D Reference Objects for organs in the HRA v2.2 alongside two United files with all organs combined (https://3d.nih.gov/collections). 22 2D FTU illustrations are at https://bioart.niaid.nih.gov/discover?collection=2. Weekly run term and relationship validation reports of ASCT+B Tables are available at https://github.com/hubmapconsortium/ccf-validation-tools/tree/master/reports. All data and SOPs are released under Creative Commons Attribution 4.0 International (CC BY 4.0).

## Box 1. Key technologies used for constructing, deploying, and using the HRA KG.

- **3D Reference Objects:** Mesh-based 3D models describing organs in the male or female body of the HRA. They are used in HRA applications to register and explore tissue blocks and associated datasets^19^. All 71 3D Reference Objects of HRA v2.2 have crosswalks that link individual 3D AS to ontology terms in Uberon or Foundational Model of Anatomy (FMA)^20,21^.
- **ASCT+B tables:** Anatomical structures (AS), cell types (CT), plus biomarkers (B) form ASCT+B tables, which are authored by multiple experts across many consortia. They capture the relationship between AS (and the AS located in them), CTs found inside these AS, and the Bs (genes, proteins) used to characterize the CT, see details in related publications^1,22^.
- **Cell Type Annotation (CTann):** Azimuth^23^, CellTypist^24,25^, and popV^26^ are used to assign cell types to cells from sc/snRNA-seq studies. Manually compiled crosswalks are used to assign ontology IDs to CTann cell types, see details in a related publication^1^.
- **Common Coordinate Framework (CCF) Ontology**: The CCF ontology^8^ provides the main vocabulary for constructing atlases of the human body, including the HRA. Critically, the CCF provides the framework for constructing atlases, but is not an atlas itself. It includes concepts and properties needed to describe the human body, from organs down to cell types and biomarkers, for organizing spatial data, and for capturing donor, sample and dataset metadata published in the KG.
- **Content negotiation**: This mechanism is used by HTTP servers to serve different versions of a resource at the same Uniform Resource Identifier (URI) based on the parameters given in the HTTP request (https://developer.mozilla.org/en-US/docs/Web/HTTP/Content_negotiation). HTTP requests can specify headers which provide additional information for the server to act on. Using the *Accept* header, an agent can specify what format (or a ranked list of acceptable formats) they would like the response returned in. A web browser will typically request *text/html*, but machines or programmers may request other formats like *application/json* or any of the RDF formats supported by the HRA KG (see subsection on Deployment in Methods > Data Processing Pipeline).
- **Crosswalks:** An ontological mapping of terms in HRA DOs to ontology terms in the ASCT+B tables^22^. Crosswalks can link, e.g., 2/3D Reference Objects of organs to AS and CT, and OMAPs^27^ to CT and B. This definition is adapted from a related publication^1^.
- **HRA Digital Objects (DOs):** HRA DOs are the data components for generating the HRA KG. They are explained in detail in the Results > HRA Digital Objects section. ASCT+B tables, 3D Reference Objects, and OMAPs are examples of HRA DOs that are processed to become part of the HRA KG.
- **Linked Open Data (LOD)**: A common data sharing pattern^28^, developed for the Semantic Web (https://www.w3.org/2001/sw/wiki/Main_Page) that describes how to structure and share semantically rich data that allows for maximum reuse and utility. To be LOD, the data should have an open license, use URIs in the data to name entities whose URIs resolve (i.e., can be queried either directly via web request or via SPARQL) to retrieve structured data in RDF about that URI and link to other resources via URIs.
- **Linked Data Modeling Language (LinkML)**: LinkML (https://linkml.io/linkml/)^29^ is a flexible linked data modeling language that allows us to author schemas in YAML (https://yaml.org) which describe the structure of one’s data. Additionally, it is a framework for working with and validating data in a variety of formats (JavaScript Object Notation [JSON], RDF, tab-separated values [TSV]), with generators for compiling LinkML schemas to other frameworks.
- **Organ Mapping Antibody Panels (OMAPs)**: Tabular data structures with panels of experiment-derived and tested antibodies to target proteins for identifying AS, CTs, cell states, or cell membrane staining in organs.
- **Persistent Uniform Resource Locator (PURL)**: A PURL is a type of URL pointing to a resolution service rather than to a website. This enables the resolution service to use content negotiation to determine what content is needed (e.g.,HTML for humans, structured data for machines) and to redirect or directly return the relevant data. PURLs are used in LOD to provide persistent, resolvable URIs for entities so that they can be referenced without worry of the URIs changing.
- **Resource Description Framework (RDF)**: RDF is a standard to represent connected data on the web. It defines relationships between data objects, enabling exchange of structured information through triples consisting of a subject, predicate, and object (https://www.w3.org/RDF/).
- **SPARQL**: A query language for RDF graphs (https://www.w3.org/TR/sparql11-query), SPARQL can be used to write declarative code to retrieve triples that describe two entities and their relationship in an RDF graph. Triples have a subject-predicate-object relationship.
- **Subject matter experts (SMEs)**: Individuals who possess specialized training in areas related to HRA construction, such as anatomists, surgeons, clinicians, and physicians. SMEs may have valuable knowledge about individual organs or organ systems or parts thereof, such as their cellular or molecular make-up, or be experts on experimental procedures.

## References

[1] Börner K. Human BioMolecular Atlas Program (HuBMAP): 3D Human Reference Atlas Construction and Usage. bioRxiv 2024.03.27.587041 (2024) doi:10.1101/2024.03.27.587041. PMC11978508 40082611

[2] Yu C. & Li. Building commonsense knowledge graphs to aid product recommendation. Amazon Science https://www.amazon.science/blog/building-commonsense-knowledge-graphs-to-aid-product-recommendation (2024).

[3] Mungall C. J., Torniai C., Gkoutos G. V., Lewis S. E. & Haendel M. A. Uberon, an integrative multi-species anatomy ontology. Genome Biol 13, R5 (2012). 22293552 10.1186/gb-2012-13-1-r5 PMC3334586

[4] Diehl A. D. The Cell Ontology 2016: enhanced content, modularization, and ontology interoperability. J. Biomed. Semant. 7, 44 (2016). 10.1186/s13326-016-0088-7 PMC4932724 27377652

[5] He Y. O. Ontology-based modeling, integration, and analysis of heterogeneous clinical, pathological, and molecular kidney data for precision medicine. 2024.04.01.587658 Preprint at 10.1101/2024.04.01.587658 (2024). PMC12099421 40417545

[6] Himmelstein D. S. Systematic integration of biomedical knowledge prioritizes drugs for repurposing. eLife 6, e26726 (2017). 28936969 10.7554/eLife.26726 PMC5640425

[7] El-Achkar T. M. A multimodal and integrated approach to interrogate human kidney biopsies with rigor and reproducibility: guidelines from the Kidney Precision Medicine Project. Physiol. Genomics 53, 1–11 (2021). 33197228 10.1152/physiolgenomics.00104.2020 PMC7847045

[8] Herr B. W. Specimen, biological structure, and spatial ontologies in support of a Human Reference Atlas. Sci. Data 10, 171 (2023). 36973309 10.1038/s41597-023-01993-8 PMC10043028

[9] Bodenreider O. The Unified Medical Language System (UMLS): integrating biomedical terminology. Nucleic Acids Res. 32, D267 (2004). 14681409 10.1093/nar/gkh061 PMC308795

[10] Stear Benjamin J. Petagraph: A large-scale unifying knowledge graph framework for integrating biomolecular and biomedical data. bioRxiv 2023.02.11.528088 (2023) doi:10.1101/2023.02.11.528088. PMC11655564 39695169

[11] Jain S. Advances and prospects for the Human BioMolecular Atlas Program (HuBMAP). Nat. Cell Biol. 25, 1089–1100 (2023). 37468756 10.1038/s41556-023-01194-w PMC10681365

[12] Snyder M. P. The human body at cellular resolution: the NIH Human Biomolecular Atlas Program. Nature 574, 187–192 (2019). 31597973 10.1038/s41586-019-1629-x PMC6800388

[13] Morris J. H. The scalable precision medicine open knowledge engine (SPOKE): a massive knowledge graph of biomedical information. Bioinforma. Oxf. Engl. 39, btad080 (2023). 10.1093/bioinformatics/btad080 PMC9940622 36759942

[14] Baranzini S. E. A biomedical open knowledge network harnesses the power of AI to understand deep human biology. AI Mag. 43, 46–58 (2022). 36093122 10.1002/aaai.12037 PMC9456356

[15] Lobentanzer S. Democratizing knowledge representation with BioCypher. Nat. Biotechnol. 41, 1056–1059 (2023). 37337100 10.1038/s41587-023-01848-y

[16] Wilkinson M. D. The FAIR Guiding Principles for scientific data management and stewardship. Sci. Data 3, 160018 (2016). 26978244 10.1038/sdata.2016.18 PMC4792175

[17] Jain Y. Segmenting functional tissue units across human organs using community-driven development of generalizable machine learning algorithms. Nat. Commun. 14, 4656 (2023). 37537179 10.1038/s41467-023-40291-0 PMC10400613

[18] Hausenblas Michael. 5-star Open Data. http://5stardata.info/en/ (2024).

[19] Börner K. Tissue registration and exploration user interfaces in support of a human reference atlas. Commun. Biol. 5, 1369 (2022). 36513738 10.1038/s42003-022-03644-x PMC9747802

[20] Golbreich C., Grosjean J. & Darmoni S. J. The Foundational Model of Anatomy in OWL 2 and its use. Artif. Intell. Med. 57, 119–132 (2013). 23273493 10.1016/j.artmed.2012.11.002

[21] Rosse C. & Mejino J. L. V. A reference ontology for biomedical informatics: the Foundational Model ofAnatomy. J. Biomed. Inform. 36, 478–500 (2003). 14759820 10.1016/j.jbi.2003.11.007

[22] Börner K. Anatomical structures, cell types and biomarkers of the Human Reference Atlas. Nat. Cell Biol. 23, 1117–1128 (2021). 34750582 10.1038/s41556-021-00788-6 PMC10079270

[23] Hao Y. Integrated analysis of multimodal single-cell data. Cell 184, 3573–3587.e29 (2021). 34062119 10.1016/j.cell.2021.04.048 PMC8238499

[24] Domínguez Conde C. Cross-tissue immune cell analysis reveals tissue-specific features in humans. Science 376, eabl5197 (2022). 35549406 10.1126/science.abl5197 PMC7612735

[25] Xu C. Automatic cell-type harmonization and integration across Human Cell Atlas datasets. Cell 186, 5876–5891.e20 (2023). 38134877 10.1016/j.cell.2023.11.026

[26] Ergen C. Consensus prediction of cell type labels in single-cell data with popV. Nat. Genet. (2024) doi:10.1038/s41588-024-01993-3. PMC11631762 39567746

[27] Quardokus E. M. Organ Mapping Antibody Panels: a community resource for standardized multiplexed tissue imaging. Nat. Methods 20, 1174–1178 (2023). 37468619 10.1038/s41592-023-01846-7 PMC10406602

[28] Berners-Lee T. Linked Data - Design Issues. https://www.w3.org/DesignIssues/LinkedData.html (2006).

[29] Moxon S. The Linked Data Modeling Language (LinkML): A General-Purpose Data Modeling Framework Grounded in Machine-Readable Semantics. in CEUR Workshop Proceedings vol. 3073 148–151 (2021).

[30] Färber M., Lamprecht D., Krause J., Aung L. & Haase P. SemOpenAlex: The Scientific Landscape in 26 Billion RDF Triples. in The Semantic Web – ISWC 2023 (eds. Payne T. R.) vol. 14266 94–112 (Springer Nature Switzerland, Cham, 2023).

[31] Jaradeh M. Y., Oelen A., Prinz M., Stocker M. & Auer S. Open Research Knowledge Graph: A System Walkthrough. in Digital Libraries for Open Knowledge: 23rd International Conference on Theory and Practice of Digital Libraries, TPDL 2019, Oslo, Norway, September 9–12, 2019, Proceedings 348–351 (Springer-Verlag, Berlin, Heidelberg, 2019). doi:10.1007/978-3-030-30760-8_31.

[32] Xu J. Building a PubMed knowledge graph. Sci. Data 7, 205 (2020). 32591513 10.1038/s41597-020-0543-2 PMC7320186

[33] Torvik V. I. MapAffil: A Bibliographic Tool for Mapping Author Affiliation Strings to Cities and TheirGeocodes Worldwide. -Lib Mag. Mag. Digit. Libr. Forum 21, (2015). 10.1045/november2015-torvik PMC4861081 27170830

[34] Cavalleri E. An ontology-based knowledge graph for representing interactions involving RNA molecules. Sci. Data 11, 906 (2024). 39174566 10.1038/s41597-024-03673-7 PMC11341713

[35] Wood E. C. RTX-KG2: a system for building a semantically standardized knowledge graph for translational biomedicine. BMC Bioinformatics 23, 400 (2022). 36175836 10.1186/s12859-022-04932-3 PMC9520835

[36] Kilicoglu H., Shin D., Fiszman M., Rosemblat G. & Rindflesch T. C. SemMedDB: a PubMed-scalerepository of biomedical semantic predications. Bioinformatics 28, 3158–3160 (2012). 23044550 10.1093/bioinformatics/bts591 PMC3509487

[37] Mendez D. ChEMBL: towards direct deposition of bioassay data. Nucleic Acids Res. 47, D930–D940 (2019). 30398643 10.1093/nar/gky1075 PMC6323927

[38] Milacic M. The Reactome Pathway Knowledgebase 2024. Nucleic Acids Res. 52, D672–D678 (2024). 37941124 10.1093/nar/gkad1025 PMC10767911

[39] Fabregat A. Reactome graph database: Efficient access to complex pathway data. PLOS Comput. Biol. 14, e1005968 (2018). 29377902 10.1371/journal.pcbi.1005968 PMC5805351

[40] Wishart D. S. DrugBank: a comprehensive resource for in silico drug discovery and exploration. Nucleic Acids Res. 34, D668–D672 (2006). 16381955 10.1093/nar/gkj067 PMC1347430

[41] Miron L., Gonçalves R. S. & Musen M. A. Obstacles to the reuse of study metadata in ClinicalTrials.gov. Sci. Data 7, 443 (2020). 33339830 10.1038/s41597-020-00780-z PMC7749162

[42] Musen M. A. The center for expanded data annotation and retrieval. J. Am. Med. Inform. Assoc. 22, 1148–1152 (2015). 26112029 10.1093/jamia/ocv048 PMC5009916

[43] O’Connor M. J. Unleashing the value of Common Data Elements through the CEDAR Workbench. AMIA. Annu. Symp. Proc. 2019, 681–690 (2020). 32308863 PMC7153094

[44] Caron A. R. A general strategy for generating expert-guided, simplified views of ontologies. 2024.12.13.628309 Preprint at 10.1101/2024.12.13.628309 (2024). PMC12827952 41513678

[45] Surles-Zeigler M. C. Extending and using anatomical vocabularies in the stimulating peripheral activity to relieve conditions project. Front. Neuroinformatics 16, (2022). 10.3389/fninf.2022.819198 PMC9449460 36090663

[46] Imam F. T. Developing a Multiscale Neural Connectivity Knowledgebase of the Autonomic Nervous System. 2024.10.25.620360 Preprint at 10.1101/2024.10.25.620360 (2024). PMC11949889 40162160

[47] SenNet Consortium NIH SenNet Consortium to map senescent cells throughout the human lifespan to understand physiological health. Nat. Aging 2, 1090–1100 (2022). 36936385 10.1038/s43587-022-00326-5 PMC10019484

[48] Dekker J. The 4D nucleome project. Nature 549, 219–226 (2017). 28905911 10.1038/nature23884 PMC5617335

[49] Lonsdale J. The Genotype-Tissue Expression (GTEx) project. Nat. Genet. 45, 580–585 (2013). 23715323 10.1038/ng.2653 PMC4010069

[50] Quardokus E. M., Record E. & Herr II B. W. SOP: Authoring Anatomical Structures, Cell Types and Biomarkers (ASCT+B) Tables. (2022) doi:10.5281/ZENODO.5746152.

[51] Bajema R. Creating 2D Illustrations for Functional Tissue Units (FTUs). (2022) doi:10.5281/zenodo.7409575.

[52] Schlehlein H. & Quardokus E. M. SOP: Creating 3D Models from Datasets. (2022) doi:10.5281/zenodo.7384276.

[53] Quardokus E. M., Bueckle A., Borner K., Record E. & Browne K. SOP: 3D Reference Object Approval. (2022) doi:10.5281/zenodo.5944197.

[54] Ju Y. & Jain Y. SOP: Computing Cell Type to Vasculature Distance Distributions. (2023) doi:10.5281/ZENODO.10371473.

[55] Weber G. & Gustilo K. Authoring the Blood Vasculature Geometry Table. (2024) doi:10.5281/zenodo.11623223.

[56] Weber G. & Gustilo K. Authoring the Pathway Organ Crosswalk Tables. (2024) doi:10.5281/zenodo.11623745.

[57] Weber G. & Gustilo K. Constructing Blood Vasculature-Organ Crosswalk Diagrams. (2024) doi:10.5281/zenodo.11623898.

[58] Kienle P., Quardokus E. M. & Bueckle A. Constructing a Millitome and Generating Virtual Tissue Blocks.(2023) doi:10.5281/ZENODO.7901004.

[59] Radtke A. J. & Quardokus E. M. SOP: Construction of Organ Mapping Antibody Panels for MultiplexedAntibody-Based Imaging of Human Tissues. Preprint at 10.5281/zenodo.5749883 (2021).

[60] McMahon A. P. GUDMAP: The Genitourinary Developmental Molecular Anatomy Project. J. Am. Soc. Nephrol. 19, 667 (2008). 18287559 10.1681/ASN.2007101078

[61] Rozenblatt-Rosen O., Stubbington M. J. T., Regev A. & Teichmann S. A. The Human Cell Atlas: fromvision to reality. Nature 550, 451–453 (2017). 29072289 10.1038/550451a

[62] The Human Tumor Atlas Network (HTAN): exploring tumor evolution in time and space. Nature https://www.nature.com/collections/fihchcjehc (2024).

[63] Bidanta S. Functional Tissue Units in the Human Reference Atlas. bioRxiv 2023.10.16.562593 (2023) doi:10.1101/2023.10.16.562593. PMC11814273 39934102

[64] Seal R. L. Genenames.org: the HGNC resources in 2023. Nucleic Acids Res. 51, D1003–D1009 (2023). 36243972 10.1093/nar/gkac888 PMC9825485

[65] Kong Y. & Börner K. Publication, funding, and experimental data in support of Human Reference Atlasconstruction and usage. Sci. Data 11, 574 (2024). 38834597 10.1038/s41597-024-03416-8 PMC11150433

[66] Bueckle A. The HRA Organ Gallery affords immersive superpowers for building and exploring the Human Reference Atlas with virtual reality. Front. Bioinforma. 3, (2023). 10.3389/fbinf.2023.1162723 PMC10174312 37181487

[67] Wolf F. A., Angerer P. & Theis F. J. SCANPY: large-scale single-cell gene expression data analysis. Genome Biol. 19, 15 (2018). 29409532 10.1186/s13059-017-1382-0 PMC5802054

[68] Harris C. R. Array programming with NumPy. Nature 585, 357–362 (2020). 32939066 10.1038/s41586-020-2649-2 PMC7759461

[69] Virshup Isaac, Rybakov Sergei, Theis Fabian J., Angerer Philipp, & Alexander Wolf F.. anndata: Annotateddata. bioRxiv 2021.12.16.473007 (2021) doi:10.1101/2021.12.16.473007.

[70] Martin F. J. Ensembl 2023. Nucleic Acids Res. 51, D933–D941 (2023). 36318249 10.1093/nar/gkac958 PMC9825606

[71] Tan S. Z. K. Brain Data Standards - A method for building data-driven cell-type ontologies. Sci. Data 10, 50 (2023). 36693887 10.1038/s41597-022-01886-2 PMC9873614

[72] Ma J. Segment Anything in Medical Images and Videos: Benchmark and Deployment. Preprint at 10.48550/arXiv.2408.03322 (2024).

[73] Ma J. Segment anything in medical images. Nat. Commun. 15, 654 (2024). 38253604 10.1038/s41467-024-44824-z PMC10803759

[74] Jain Y. Segmentation of human functional tissue units in support of a Human Reference Atlas. Commun. Biol. 6, 717 (2023). 37468557 10.1038/s42003-023-04848-5 PMC10356924

[75] Wang X. (Julie) Generalized cell phenotyping for spatial proteomics with language-informed vision models. 2024.11.02.621624 Preprint at 10.1101/2024.11.02.621624 (2024).

[76] Israel U. A Foundation Model for Cell Segmentation. Preprint at 10.48550/arXiv.2311.11004 (2023). PMC12695629 41360960

[77] Jain Y. Vasculature segmentation in 3D hierarchical phase-contrast tomography images of human kidneys. 2024.08.25.609595 Preprint at 10.1101/2024.08.25.609595 (2024). PMC13470036 42399229

[78] Yagis E. Deep learning for 3D vascular segmentation in hierarchical phase contrast tomography: a case study on kidney. Sci. Rep. 14, 27258 (2024). 39516256 10.1038/s41598-024-77582-5 PMC11549215

[79] Brbić M. Annotation of spatially resolved single-cell data with STELLAR. Nat. Methods 19, 1411–1418 (2022). 36280720 10.1038/s41592-022-01651-8 PMC12186200

[80] Greenwald N. F. Whole-cell segmentation of tissue images with human-level performance using large-scale data annotation and deep learning. Nat. Biotechnol. 40, 555–565 (2022). 34795433 10.1038/s41587-021-01094-0 PMC9010346

[81] Pan H. Comprehensive anatomic ontologies for lung development: A comparison of alveolar formation and maturation within mouse and human lung. J. Biomed. Semant. 10, 18 (2019). 10.1186/s13326-019-0209-1 PMC6814058 31651362

[82] Ontology Lookup Service. Provisional Cell Ontology. https://www.ebi.ac.uk/ols4/ontologies/pcl.

[83] Weber G. M., Ju Y. & Börner K. Considerations for Using the Vasculature as a Coordinate System to MapAll the Cells in the Human Body. Front. Cardiovasc. Med. 7, (2020). 10.3389/fcvm.2020.00029 PMC7082726 32232057

[84] Cyberinfrastructure for Network Science Center. HRA Organ Gallery on Horizon Store. Oculus https://www.meta.com/experiences/quest/5696814507101529/ (2024).

