# Supplementary material for: Construction, Deployment, and Usage of the Human Reference Atlas Knowledge Graph for Linked Open Data
Source: bioRxiv. 2025 Feb 19:2024.12.22.630006. Originally published 2024 Dec 23. Preprint. [Version 2] doi: 10.1101/2024.12.22.630006 (PMC11703146; doi:10.1101/2024.12.22.630006)
Supplement: Supplement 1 [file NIHPP2024.12.22.630006v2-supplement-1.pdf]

bioRxiv preprint doi: https://doi.org/10.1101/2024.12.22.630006; this version posted February 19, 2025. The copyright holder for this preprint (which was not certified by peer review) is the author/funder, who has granted bioRxiv a license to display the preprint in perpetuity. It is made available under aCC-BY 4.0 International license.

# Supplemental Figures


```yaml
data:
  cell_marker_descriptors:
   - id: HRA:R15-desc
     label: Cell marker descriptor for Proximal Tubule  Epithelial Cell
     type_of:
      - CellMarkerDescriptor
     primary_anatomical_structure: UBERON:0004134
     primary_cell_type: CL:0002306
     biomarker_set:
      - HGNC:2548
      - HGNC:6694
      - HGNC:10916
      - HGNC:417
      - HGNC:4175
     references:
       - https://www.doi.org/10.1038/s41467-019-10861-2
       - https://www.doi.org/10.1172/jci.insight.13326
       - https://www.doi.org/10.1038/s41586-020-2941-1
       - https://www.doi.org/10.1073/pnas.2005477117
       - https://www.doi.org/10.1681/ASN.2020010071
     derived_from: https://lod.humanatlas.io/asct-b/kidney/v1.5
```


**Supplemental Figure 1.** An excerpt of a normalized structure from the ASCT+B table for the kidney, with a focus on *cell_marker_descriptors*, where primary CT, primary anatomical location, and associated characterizing B are detailed, and where references to support the claims regarding the CT and its B are provided. An example in YAML is provided at https://github.com/cns-iu/hra-kg-supporting-information/blob/main/docs/intermediary_format.yaml.

bioRxiv preprint doi: https://doi.org/10.1101/2024.12.22.630006; this version posted February 19, 2025. The copyright holder for this preprint (which was not certified by peer review) is the author/funder, who has granted bioRxiv a license to display the preprint in perpetuity. It is made available under aCC-BY 4.0 International license.

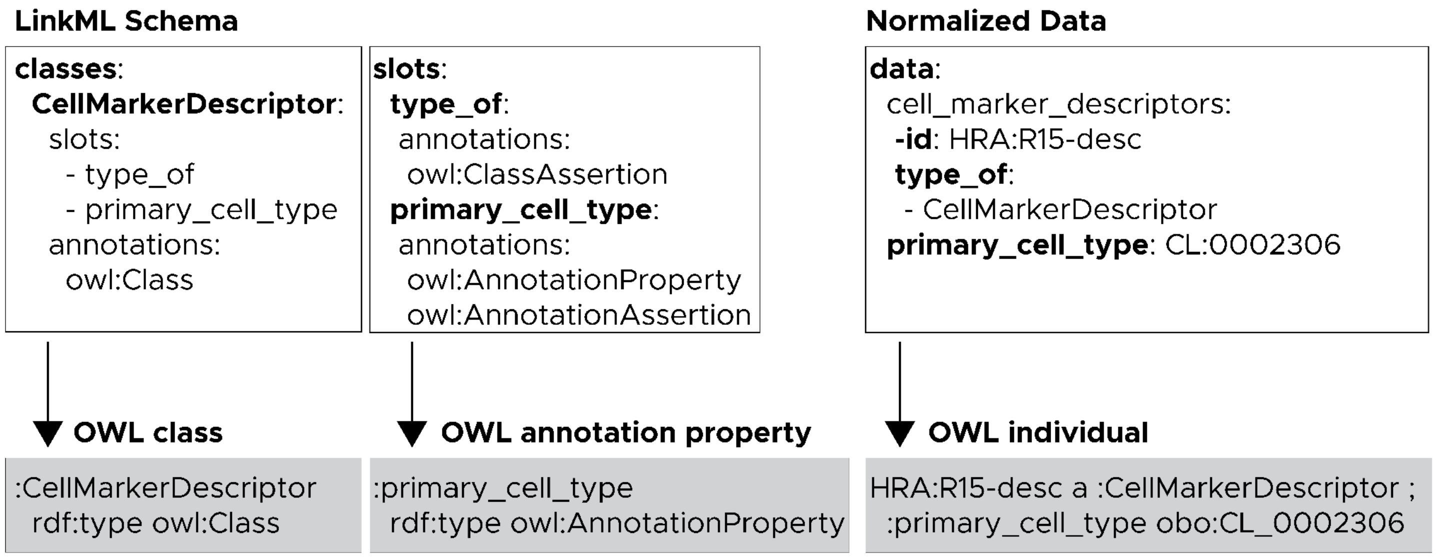


**Supplemental Figure 2.** This diagram shows an excerpt of a LinkML schema (represented by the two boxes on the left) and the corresponding acquired data (the box on the right). Arrows illustrate the transformation of the input text into OWL constructs at the bottom. In the schema boxes, classes and slots are directly mapped to OWL classes and OWL properties as specified in the *annotations* field. In the data box, each data item pair is translated into an OWL assertion statement. For example, the data pair *type_of: CellMarkerDescriptor* generates a class assertion that indicates the data object belongs to the CellMarkerDescriptor class. Similarly, the data pair *primary_cell_type: CL:0002306* produces an annotation assertion that tells the same data object identifies the epithelial cell in the proximal tubule of the kidney (CL:0002306) as the primary CT.

bioRxiv preprint doi: https://doi.org/10.1101/2024.12.22.630006; this version posted February 19, 2025. The copyright holder for this preprint (which was not certified by peer review) is the author/funder, who has granted bioRxiv a license to display the preprint in perpetuity. It is made available under aCC-BY 4.0 International license.

# Supplemental Tables

**Supplemental Table 1**. Graphs of the HRA KG (https://lod.humanatlas.io/graph).

| Name | Description | Link |
|---|---|---|
| 2d-ftu-illustrations | Contains 2D assets for FTUs. | https://lod.humanatlas.io/graph/2d-ftu-illustrations |
| ccf | Graph representation of the CCF.OWL dataset. | https://lod.humanatlas.io/graph/ccf |
| ctann-crosswalks | Graph representation of CTann crosswalks for HRApop (Azimuth[11], CellTypist[12,13] popV[14]). | https://lod.humanatlas.io/graph/ctann-crosswalks |
| ctpop | (Deprecated) version of the hra-pop *graph*, see below | https://lod.humanatlas.io/graph/ctpop |
| ds-graphs-enrichments | Enriched graph of all public dataset graphs for the HRA. | https://lod.humanatlas.io/graph/ds-graphs-enrichments |
| hra-ccf-body | Graph representation of the 3D Reference Objects for human male and female bodies. | https://lod.humanatlas.io/graph/hra-ccf-body |
| hra-ccf-patches | Graph that provides placements (patches) to convert old 3D Reference Object registrations to newer versions for reference organs that had a different shape, placement, or size in earlier versions. | https://lod.humanatlas.io/graph/hra-ccf-patches |
| hra-pop | Contains donor and sample metadata as well as 3D extraction sites and cell summaries for 619 ATLAS datasets used in HRA construction (as of v0.11.1). Code to compute mean B expression values per cell per dataset is at https://github.com/hubmapconsortium/hra-workflows/blob/3dfea9ce19138c930dec92024496c4814a290f12/containers/gene-expression/context/main.py#L108-L151<br><br>Exemplarily, the lookup table between Ensembl Release 111[34] (https://www.ensembl.org/index.html) and HGNC v2023-09-18[72] (https://www.genenames.org) for CellTypist is at https://github.com/hubmapconsortium/hra-workflows/blame/main/containers/celltypist/context/ensemble-lookup.csv | https://lod.humanatlas.io/graph/hra-pop |
| HRAlit (currently served as a relational database) | Links the HRA DOs to publications, experts, experimental datasets, and funded projects[32] to identify relevant papers, SMEs, alignment with existing ontologies, and funding trends to HRA construction and usage. | N/A |

bioRxiv preprint doi: https://doi.org/10.1101/2024.12.22.630006; this version posted February 19, 2025. The copyright holder for this preprint (which was not certified by peer review) is the author/funder, who has granted bioRxiv a license to display the preprint in perpetuity. It is made available under aCC-BY 4.0 International license.

**Supplemental Table 2**. Collections of the HRA KG (https://lod.humanatlas.io/collection). All structures are built using standardized terms for labels, which are stored in the specialized CCF vocabulary, see CCF Ontology in **Box 1**.

| Name | Description | Link |
|---|---|---|
| ds-graphs | Consolidates experiment-result datasets (*ds-graph*) into a single graph data structure. Contains processed graphs of datasets including donor and sample metadata from different consortia and tissue providers registered to HRA via 3D extraction sites, available at https://lod.humanatlas.io/ds-graph. Also present are HRA *graph* DOs (https://lod.humanatlas.io/graph), which are externally processed RDF graphs, such as crosswalks between CT assigned by CTann tools and CT in ASCT+B tables. | https://lod.humanatlas.io/co graphs |
| hra | Includes HRA DO types with a DOI. As of HRA v2.2, those are *2d-ftu*, *asct-b*, *ctann, omap*, *ref-organ*, and *vascular-geometry* DO types. Other DO types can be added in the future once they have DOIs. | https://lod.humanatlas.io/collection/hra |
| hra-api | Centers on *asct-b* and associated 3D models (*ref-organ, landmark*) used in various HRA applications for visualizing and organizing reference data. | https://lod.humanatlas.io/collection/hra-api |
| hra-ols | HRA collection subset that focuses solely on AS, CT, and B (*asct-b* only). It is used by OLS (https://www.ebi.ac.uk/ols4) for validation. The source for the files on the LOD server are available at https://github.com/hubmapconsortium/3d-hra-ref-object-validation. | https://lod.humanatlas.io/collection/hra-ols |

bioRxiv preprint doi: https://doi.org/10.1101/2024.12.22.630006; this version posted February 19, 2025. The copyright holder for this preprint (which was not certified by peer review) is the author/funder, who has granted bioRxiv a license to display the preprint in perpetuity. It is made available under aCC-BY 4.0 International license.

**Supplemental Table 3**. Most HRA applications use the HRA KG as their backend database. Also listed are external applications that use the HRA KG.

| Name | Description | URL |
|---|---|---|
| HRA Portal | Provides the landing page and additional web pages for exploring HRA-related resources. Some data statistics on the page are aggregated from the HRA KG via SPARQL queries. | https://humanatlas.io |
| RUI | Allows users to register new tissue samples and related datasets into the human reference atlas[6]. The "parts" hierarchy on the navigation panel and the 3D human model at the center of the page are generated from the HRA KG via HRA API queries. | https://apps.humanatlas.io/rui |
| EUI | Enables users to explore and query the HRA KG interactively[6]. All visualizations, data facets, statistics, and data browsers are generated through real-time querying of the knowledge graph. | https://apps.humanatlas.io/eui |
| ASCT+B Reporter | Enables users to inspect ASCT+B Tables as tree visualizations[6] and supports OMAP comparison to ASCT+B tables. Uses the HRA KG to serve processed and published ASCT+B Tables to the website. | https://apps.humanatlas.io/asctb-reporter |
| FTU Explorer | Facilitates interactive exploration of FTUs within the HRA. The HRA KG provides the *2d-ftu* DOs that the FTU Explorer displays. Datasets and cell summaries registered to these FTUs are currently separate but will be available in the HRA KG as *ds-graph* DOs in the future. | https://humanatlas.io/2d-ftu-illustrations |
| HRA Dashboard | Offers a summary and visual representation of data statistics and trends within the HRA knowledge graph. Data statistics and charts are generated by aggregating data from the HRA KG. | https://apps.humanatlas.io/dashboard |
| HRA API endpoints | Provides programmatic access to the HRA KG, enabling integration with other applications and services. A SPARQL endpoint for the HRA API allows users to write their own SPARQL queries. Canned queries are available at https://apps.humanatlas.io/api/grlc/. | API documentation: https://humanatlas.io/api |
| HRA Organ Gallery | Enables an immersive view of the HRA by showing 71 reference organs and 1,100+ tissue blocks alongside cell type populations in VR.[61,73] | https://humanatlas.io/hra-organ-gallery |
| Cell Distance Explorer | Allows distance visualizations between cells in 2D and 3D. Features histograms and violin graphs for vascular distances by CT. Does not use HRA KG but will use it for crosswalking CT to CL and PCL in the future. | https://apps.humanatlas.io/cde |
| **In Data Portals** | | |
| HuBMAP Data Portal | Same as EUI above but only showing HuBMAP data | https://portal.hubmapconsortium.org/ccf-eui |
| SenNet Data Portal | Same as EUI above but only showing SenNet data | https://data.sennetconsortium.org/ccf-eui |
| GTEx Portal | Same as EUI above but only showing GTEx data | https://gtexportal.org/home/eui |
| KPMP Tissue Atlas | Same as EUI above but only showing KPMP data | https://apps.humanatlas.io/eui/kpmp.html |

bioRxiv preprint doi: https://doi.org/10.1101/2024.12.22.630006; this version posted February 19, 2025. The copyright holder for this preprint (which was not certified by peer review) is the author/funder, who has granted bioRxiv a license to display the preprint in perpetuity. It is made available under aCC-BY 4.0 International license.

**Supplemental Table 4.** An overview of all GitHub repositories used to construct, deploy, and use the HRA KG.

| Name | Description | URL |
|---|---|---|
| **Code** | | |
| hra-kg | HRA Knowledge Graph | https://github.com/hubmapconsortium/hra-kg |
| hra-api | HRA API | https://github.com/x-atlas-consortia/hra-api |
| hra-do-processor | HRA DO Processor | https://github.com/hubmapconsortium/hra-do-processor |
| ccf-grlc | Repository of canned SPARQL queries that can be run like a REST API endpoint via https://grlc.io | https://github.com/hubmapconsortium/ccf-grlc |
| hra-ubkg-exporter | A CLI for exporting a subset of the HRA KG to Unified Biomedical Knowledge Graph (UBKG) format | https://github.com/x-atlas-consortia/hra-ubkg-exporter |
| hra-kg-releases/hra-kg.v2.2.tar.xz | Whole HRA KG, ~5.1GB. | https://cdn.humanatlas.io/hra-kg-releases/hra-kg.v2.2.tar.xz |
| **APIs and Documentation** | | |
| HRA KG SPARQL endpoint | SPARQL endpoint of the HRA KG | https://lod.humanatlas.io/sparql |
| HRA API | UI with documentation for all HRA API endpoints and routes | https://apps.humanatlas.io/api |
| HRA API SPARQL queries | Canned SPARQL queries via grlc.io on HRA API | https://apps.humanatlas.io/api/grlc/ |

bioRxiv preprint doi: https://doi.org/10.1101/2024.12.22.630006; this version posted February 19, 2025. The copyright holder for this preprint (which was not certified by peer review) is the author/funder, who has granted bioRxiv a license to display the preprint in perpetuity. It is made available under aCC-BY 4.0 International license.

**Supplemental Table 5**. List of LinkML schemas in the HRA KG.

| Schema Description | Link |
|---|---|
| Graph schema for ASCT+B tables | https://github.com/hubmapconsortium/hra-do-processor/blob/main/schemas/src/digital-objects/asct-b.yaml |
| Graph schema for 3D Reference Objects for organs | https://github.com/hubmapconsortium/hra-do-processor/blob/main/schemas/src/digital-objects/ref-organ.yaml |
| Graph schema for 2D FTUs | https://github.com/hubmapconsortium/hra-do-processor/blob/main/schemas/src/digital-objects/2d-ftu.yaml |
| Graph schema for the 3D Landmarks | https://github.com/hubmapconsortium/hra-do-processor/blob/main/schemas/src/digital-objects/landmark.yaml |
| Graph schema for OMAPs | https://github.com/hubmapconsortium/hra-do-processor/blob/main/schemas/src/digital-objects/omap.yaml |
| Graph schema for experimental dataset metadata | https://github.com/hubmapconsortium/hra-do-processor/blob/main/schemas/src/digital-objects/ds-graph.yaml |
| Graph schema for external graph files (metadata only) | https://github.com/hubmapconsortium/hra-do-processor/blob/main/schemas/src/digital-objects/graph.yaml |
| Graph schema for vocabulary resources (metadata only) | https://github.com/hubmapconsortium/hra-do-processor/blob/main/schemas/src/digital-objects/vocab.yaml |
| Graph schema for curated graph collections (metadata only) | https://github.com/hubmapconsortium/hra-do-processor/blob/main/schemas/src/digital-objects/collection.yaml |
| Graph schema for cell summaries | https://github.com/hubmapconsortium/hra-do-processor/blob/main/schemas/src/modules/cell-summary.yaml |
| Graph schema for spatial data | https://github.com/hubmapconsortium/hra-do-processor/blob/main/schemas/src/modules/spatial.yaml |
| Graph schema for spatial collision data | https://github.com/hubmapconsortium/hra-do-processor/blob/main/schemas/src/modules/collision.yaml |
| Graph schema for spatial corridor data | https://github.com/hubmapconsortium/hra-do-processor/blob/main/schemas/src/modules/corridor.yaml |
| Graph schema for assay dataset metadata | https://github.com/hubmapconsortium/hra-do-processor/blob/main/schemas/src/modules/dataset.yaml |
| Graph schema for donor metadata. | https://github.com/hubmapconsortium/hra-do-processor/blob/main/schemas/src/modules/donor.yaml |
| Graph schema for sample metadata | https://github.com/hubmapconsortium/hra-do-processor/blob/main/schemas/src/modules/sample.yaml |

bioRxiv preprint doi: https://doi.org/10.1101/2024.12.22.630006; this version posted February 19, 2025. The copyright holder for this preprint (which was not certified by peer review) is the author/funder, who has granted bioRxiv a license to display the preprint in perpetuity. It is made available under aCC-BY 4.0 International license.

| | |
|---|---|
| Graph schema for defining entities | https://github.com/hubmapconsortium/hra-do-processor/blob/main/schemas/src/shared/entity-base.yaml |
| Graph schema for defining instances | https://github.com/hubmapconsortium/hra-do-processor/blob/main/schemas/src/shared/instance-base.yaml |
| Graph schema for defining graph metadata | https://github.com/hubmapconsortium/hra-do-processor/blob/main/schemas/src/shared/metadata-base.yaml |

bioRxiv preprint doi: https://doi.org/10.1101/2024.12.22.630006; this version posted February 19, 2025. The copyright holder for this preprint (which was not certified by peer review) is the author/funder, who has granted bioRxiv a license to display the preprint in perpetuity. It is made available under aCC-BY 4.0 International license.

**Supplemental Table 6.** Publications on HRA DOs plus aliases used throughout HRA publications and applications.

| HRA DO Type | Aliases | Publication Title | DOI |
|---|---|---|---|
| 2d-ftu | - Functional Tissue Units<br>- Functional Tissue Units (FTUs)<br>- FTUs | Functional Tissue Units in the Human Reference Atlas | https://doi.org/10.1101/2023.10.16.562593 |
| asct-b | - Anatomical Structures, Cell Types, and Biomarker Tables<br>- Anatomical Structures, Cell Types, and Biomarker Tables (ASCT+B) Tables<br>- ASCT+B Tables | Anatomical structures, cell types and biomarkers of the Human Reference Atlas | https://www.nature.com/articles/s41556-021-00788-6 |
| | | Anatomical structures, cell types, and biomarkers of the healthy human blood vasculature | https://www.nature.com/articles/s41597-023-02018-0 |
| ctann | - Cell Type Annotations<br>- CTann | | |
| landmark | - Landmark Organs<br>- Landmark Anatomical Structures | | |
| millitome | - Millitome | | |
| omap | - Organ Mapping Antibody Panels<br>- Organ Mapping Antibody Panels (OMAPs)<br>- OMAPs<br>- Organ Mapping Antibody Panel Tables | Organ Mapping Antibody Panels: a community resource for standardized multiplexed tissue imaging | https://www.nature.com/articles/s41592-023-01846-7 |
| ref-organ | - 3D Reference Object<br>- 3D Reference Organ | Tissue registration and exploration user interfaces in support of a human reference atlas | https://www.nature.com/articles/s42003-022-03644-x |
| schema | - Schema | | |
| vascular-geometry | | Anatomical structures, cell types, and biomarkers of the healthy human blood vasculature | https://www.nature.com/articles/s41597-023-02018-0 |
| | | Considerations for Using the Vasculature as a Coordinate System to Map All the Cells in the Human Body | https://doi.org/10.3389/fcvm.2020.00029 |
| vocab | | Specimen, biological structure, and spatial ontologies in support of a Human Reference Atlas | https://www.nature.com/articles/s41597-023-01993-8 |

bioRxiv preprint doi: https://doi.org/10.1101/2024.12.22.630006; this version posted February 19, 2025. The copyright holder for this preprint (which was not certified by peer review) is the author/funder, who has granted bioRxiv a license to display the preprint in perpetuity. It is made available under aCC-BY 4.0 International license.

**Supplemental Table 7**. HRA User Stories and how the HRA KG supports them.

| HRA User Story | Value Added by HRA KG | HRA KG Sample Queries |
|---|---|---|
| **US#1.** Predict cell type populations | Use the HRA KG to access the hra-pop *graph* (https://lod.humanatlas.io/graph/hra-pop/latest) with cell type populations for AS, datasets, and extraction sites to improve the accuracy of annotations for sc-transcriptomics and sc-proteomics datasets. In the future, the HRA KG could be used to store metadata for millions of cells individually. | /as-weighted-cell-summaries: https://github.com/x-atlas-consortia/hra-api/blob/main/src/library/hra-pop/queries/as-weighted-cell-summaries.rq<br><br>Accessible via HRA API: https://apps.humanatlas.io/api/hra-pop/rui-location-cell-summary |
| **US#2.** Predict spatial origin of tissue samples | Use the cell type populations from the hra-pop *graph* mentioned above to predict the 3D location of datasets with unknown spatial origin. | /select-cell-summaries: https://github.com/x-atlas-consortia/hra-api/blob/main/src/library/hra-pop/queries/select-cell-summaries.rq<br><br>Accessible via HRA API: https://apps.humanatlas.io/api/hra-pop/cell-summary-report<br><br>/supported-organs: https://github.com/x-atlas-consortia/hra-api/blob/main/src/library/hra-pop/queries/supported-organs.rq<br><br>/supported-reference-organs: https://github.com/x-atlas-consortia/hra-api/blob/main/src/library/hra-pop/queries/supported-reference-organs.rq<br><br>/supported-tools: https://github.com/x-atlas-consortia/hra-api/blob/main/src/library/hra-pop/queries/supported-tools.rq |
| **US#3.** Compare reference tissue with aging/diseased tissue | Use the EUI to examine AS, CT, and B. Via the HRA API, the RUI runs SPARQL queries of the HRA KG to retrieve the AS partonomy, CT typology, and B. | /tissue-blocks: https://apps.humanatlas.io/api/#get-/v1/tissue-blocks (then user parameters to get datasets by age (range), BMI (range), sex<br><br>Accessible via HRA API: https://apps.humanatlas.io/api/#get-/v1/tissue-blocks (then user parameters to get datasets by age (range), BMI (range), sex<br><br>/scene: https://github.com/x-atlas-consortia/hra-api/blob/main/src/library/v1/queries/scene.rq Note: #{{FILTER}} gets replaced with filters documented on HRA API endpoint at https://apps.humanatlas.io/api/#get-/v1/scene to get datasets by age (range), BMI (range), sex, etc. |
| **US#4.** Compare reference FTUs with | Use the HRA KG to retrieve 2D illustrations of 22 FTUs in the FTU Explorer | /ftu-parts: https://apps.humanatlas.io/api/grlc/hra.html# |

bioRxiv preprint doi: https://doi.org/10.1101/2024.12.22.630006; this version posted February 19, 2025. The copyright holder for this preprint (which was not certified by peer review) is the author/funder, who has granted bioRxiv a license to display the preprint in perpetuity. It is made available under aCC-BY 4.0 International license.

| | | |
|---|---|---|
| aging/diseased FTUs | (https://apps.humanatlas.io/ftu-explorer/#/) | get-/ftu-parts<br><br>/2d-ftu-illustrations (DO) https://lod.humanatlas.io/graph/2d-ftu-illustrations |
| **US#5.** Provide cell distance distribution visualizations | Use the Cell Distance Explorer to visualize distance distributions between different cells and CT in 2D or 3D sc-proteomics tissue. In the future, the HRA KG will be used to crosswalk CT labels to CL (https://obofoundry.org/ontology/cl.html)[19] to add automated grouping features. Cell distances could also be stored in the HRA KG. | No sample queries yet, but support is possible for adding comparisons to other datasets or simpler color scheme via CL once crosswalks exist |
| **US#6.** Develop lightweight atlas components | Use the HRA KG to enable HRA web components to improve data access and analysis outside of the HRA applications ecosystem (https://apps.humanatlas.io/us6/). More information is provided under Results > Using the HRA KG > HRA Applications. | Components use HRA KG, e.g., EUI component uses HRA KG via HRA API, same for RUI and FTU Explorer (see US#3 and 4) |
| **US#7.** Implement dashboard for HRA | Explore usage statistics of atlas data and code to check HRA growth and outreach over time. SPARQL queries to the HRA KG serve stats on experimental data and all HRA DO types | /digital-objects-per-organ: https://github.com/x-atlas-consortia/hra-dashboard-data/blob/main/queries/sparql/data/digital-objects-per-organ.rq<br><br>/as-per-organ: https://github.com/x-atlas-consortia/hra-dashboard-data/blob/main/queries/sparql/humanatlas.io/as-per-organ.rq<br><br>hra-growth.atlas.rq https://github.com/x-atlas-consortia/hra-dashboard-data/blob/main/queries/sparql/data/hra-growth.atlas.rq |
